# The Empirical Distribution of Singletons for Geographic Samples of DNA Sequences

**DOI:** 10.3389/fgene.2017.00139

**Published:** 2017-09-29

**Authors:** Philippe Cubry, Yves Vigouroux, Olivier François

**Affiliations:** ^1^UMR DIADE, University of Montpellier, Montpellier, France; ^2^TIMC-IMAG UMR 5525, Centre National de la Recherche Scientifique (CNRS), Université Grenoble-Alpes, Grenoble, France

**Keywords:** genetic diversity, singletons, geographic origin, range expansion, pearl millet

## Abstract

Rare variants are important for drawing inference about past demographic events in a species history. A singleton is a rare variant for which genetic variation is carried by a unique chromosome in a sample. How singletons are distributed across geographic space provides a local measure of genetic diversity that can be measured at the individual level. Here, we define the empirical distribution of singletons in a sample of chromosomes as the proportion of the total number of singletons that each chromosome carries, and we present a theoretical background for studying this distribution. Next, we use computer simulations to evaluate the potential for the empirical distribution of singletons to provide a description of genetic diversity across geographic space. In a Bayesian framework, we show that the empirical distribution of singletons leads to accurate estimates of the geographic origin of range expansions. We apply the Bayesian approach to estimating the origin of the cultivated plant species *Pennisetum glaucum [L.] R. Br*. (pearl millet) in Africa, and find support for range expansion having started from Northern Mali. Overall, we report that the empirical distribution of singletons is a useful measure to analyze results of sequencing projects based on large scale sampling of individuals across geographic space.

## 1. Introduction

High-throughput sequencing technologies have enabled studies of genomic diversity in model and non-model species at a dramatically increasing rate. Conducted at population and at individual levels, those studies have provided comprehensive surveys of common and rare variation in model species genomes (Weigel and Mott, 2009; 1000 Genomes Project Consortium et al., 2010; International HapMap 3 Consortium, 2010; 1000 Genomes Project Consortium, 2015). For example, the 1000 Genomes Project Consortium (2015) reported that the majority of variants in human genomes are rare. During the last decade, the role that rare variants play in shaping complex traits has been hotly debated (Pritchard, 2001; Schork et al., 2009; Tennessen et al., 2012), and accurately determining their distribution has become important for medical applications and association studies (Lee et al., 2014; Auer and Lettre, 2015). Beyond humans, rare variation has attracted considerable interest from genome sequencing projects for model organisms, including plants (Zhu et al., 2011; Weigel, 2012; Memon et al., 2016).

Rare variants are also important for drawing inference about past demographic events in a species history (Schraiber and Akey, 2015). Studies of human populations have shown that our species has experienced a complex demographic history, and that a recent period of explosive growth has resulted in an excess of those variants (Coventry et al., 2010; Keinan and Clark, 2012). The analysis of private and rare variation has been used to reveal signals of differential demographic history among populations, and to refine models of human evolution (Marth et al., 2004; Gravel et al., 2011; Mathieson and McVean, 2014). In addition, estimating rare allele frequencies has enabled estimates of gene flow between populations, and has facilitated inference of fine-scale population structure (Slatkin, 1985; Novembre and Slatkin, 2009; O'Connor et al., 2015).

In this study, we define the empirical distribution of singletons in a sample of chromosomes as the proportion of the total number of singletons that each chromosome carries, where a singleton is a uniquely represented allele in the sample (Fu and Li, 1993). We provide theoretical and empirical analyses of the distribution of singletons in a sample of chromosomes, and we evaluate the potential for this distribution to provide an accurate description of genetic diversity at the individual level. Using spatial data, we use the distribution of singletons as an individual-based estimate of genetic diversity in geographic space.

The theoretical background for the analysis of the empirical distribution of singletons rely on the distribution of external branch lengths for coalescent genealogies (Blum and François, 2005; Caliebe et al., 2007). First, we use coalescent and spatially explicit simulations to evaluate individual contributions to genetic diversity in the sample based on singletons. Then we evaluate the use of the distribution of singletons in an approximate Bayesian Computation (ABC) framework to estimate the geographic origin of range expansions (Beaumont, 2010; Csilléry et al., 2010). We eventually provide an illustration of our theory by applying the ABC approach to the plant species *Pennisetum glaucum [L.] R. Br*. (pearl millet). Pearl millet is a cereal cultivated in semi-arid regions of Africa and the Indian subcontinent, and it is known to originate in Africa (Clotault et al., 2012). We evaluate the geographic origin of its range expansion by using 146 inbred lines from the whole African range.

## 2. Theory

We consider a sample of *n* chromosomes from a population of *N* haploid organisms. We assume that there are *L* polymorphic loci, and that for each locus, 0 represents the ancestral or reference allele and 1 is the derived allele. A singleton is defined as a derived allele carried by a single chromosome in the sample. The total number of singletons, ξ_1_, is the number of uniquely represented derived alleles in the sample, and it corresponds to the first component of the site frequency spectrum. We assume that the singletons are distributed over the *n* chromosomes in the sample. More specifically, the number of singletons decomposes as follows

$$\xi_{1}=\overset{n}{\underset{i=1}{\sum }}\xi_{1}^{\left(i\right)},$$

where $\xi_{1}^{\left(i\right)}$ is the number of singletons carried by chromosome *i*. For each *i*, we denote by *p*_*i*_ the conditional probability that a singleton is carried by *i*. The *n* values *p*_1_, …, *p*_*n*_ sum up to one, and those values define the *empirical distribution of singletons* in the sample (see below).

Next, we assume that the sample genealogies can be described by coalescent trees (Tavaré, 2004). For a particular locus, a tree is described by *n* tips and *n*−1 ancestral nodes. An external branch of the tree connects a tip to an ancestral node. For a given tree, we denote by τ^(*i*)^ the length of the external branch connecting chromosome *i* to its first ancestor node. The *L* coalescent trees exhibit complex patterns of statistical dependency along the chromosomes due to recombination among loci (Hudson, 1990). Measuring lengths in units of twice the total population size (*N*), and assuming a molecular clock model for mutations, the number of mutations falling on a particular branch of the tree has a Poisson distribution of rate θ/2, where θ = 2μ*N* and μ is the per generation mutation rate (Tavaré, 2004). Let ℓ be an arbitrary singleton locus. For all *i*, we write

$$\xi_{1}^{\left(i\right)}=\overset{\xi_{1}}{\underset{k=1}{\sum }}X_{i\ell },$$

where *X*_*iℓ*_ = 1 if singleton ℓ is carried by chromosome *i*, 0 otherwise. In the above formula, the summation runs over all singletons in the sample. Using mathematical properties of conditional distributions for the Poisson process, we have

$$p_{i}=\text{P}\left(X_{i\ell }=1\right)=\text{E }\left[\frac{\tau_{1}^{\left(i\right)}}{\tau_{1}}\right],$$

where $\tau_{1}=\overset{n}{\underset{i=1}{\sum }}\tau^{\left(i\right)}$. In this formula, the conditional probability that chromosome *i* carries a singleton at locus ℓ is given by the ratio of its external branch length to the total length of external branches in the sample genealogy at this locus. The distribution of singletons can be estimated by counting the number of singletons carried by each chromosome and normalizing as follows

$${\hat{p}}_{i}=\xi_{1}^{\left(i\right)}/\xi_{1},\;i=1,\ldots ,n,$$

and the estimate is unbiased

$$\text{E}\left[{\hat{p}}_{i}\right]=p_{i}.$$

In addition, the number of singletons carried by chromosome *i*, $\xi_{1}^{\left(i\right)}$, estimates the proportion of genetic diversity carried by chromosome *i*

$$\text{E}\left[\xi_{1}^{\left(i\right)}\right]\approx \theta p_{i},\;i=1,\ldots ,n.$$

As a consequence of the theory presented in this section, the individual-based estimates of genetic diversity are unbiased quantities regardless of demographic history, deviations from Hardy-Weinberg equilibrium and linkage disequilibrium. Limitations of the theory include the presence of closely related individuals, which should be removed from the sample prior to analysis. The approach is appropriate for modern sequencing data as soon as a few hundreds of DNA sequences are generated.

The rest of this study will evaluate the use of the empirical distribution of singletons in mapping genetic diversity in geographic space. To provide an elementary example, let us consider a sample of *n* chromosomes from a random mating population of size *N*. Using mathematical results for the neutral coalescent in a random mating population, the expected value of the number of singletons is an unbiased estimator of the genetic diversity in the sample (Fu and Li, 1993)

$$\text{E}\left[\xi_{1}\right]=\theta .$$

For the lengths of external branch lengths, we have

$$\text{E}\left[\tau^{\left(i\right)}\right]=2/n,\;i=1,\ldots ,n,$$

and E[τ_1_] = 2 (Blum and François, 2005). Here, we expect that each chromosome contributes to genetic diversity equally. The above calculations show that, in a sample of size *n* from a random mating population, the distribution of singletons is uniform over the *n* chromosomes

$$p_{i}=1/n,\;i=1,\ldots ,n,$$

and we have

$$\text{E}\left[\xi_{1}^{\left(i\right)}\right]=\text{E}\left[\xi_{1}\right]\text{P}\left(X_{ik}=1\right)=\theta /n.$$

In other words, each individual contributes the same amount of genetic variation to the total sample diversity.

## 3. Simulation methods and data sets

### 3.1. Coalescent simulations of splitting populations

We used the computer program *ms* to perform coalescent simulations for a two-population model (Hudson, 2002). In our simulations, we considered a population split model, in which two populations of sizes *N*_1_ = 50, 000 and *N*_2_ = *sN*_1_ (*s* ∈ (0.01;0.5), shrink rate) diverged *t* generations ago (*t* ∈ (1, 000;10, 000), split time). Population 1 expanded from an ancestral population of size *N*_*A*_ = 5, 000, and the expansion started 10,000 generations ago. Samples of size *n* = 100 were considered and subdivided into subsamples of size 50 from each population. We simulated *L* = 1, 000 unlinked haplotypes using the infinite-site model and an effective mutation rate θ ∈ (5;10). The *ms* command line was written as follows: ./ms 100 1,000 -t theta -I 2 50 50 -g 1 46.05 -n 2 shrink.rate -eg 0.2 1 0.0 -ej split.time 2 1. The simulated data sets were processed by using the “.geno” format in the R package LEA (Frichot and François, 2015). We summarized the distribution of singletons by computing mean values and standard errors for each subsample. For all simulated samples, we used the R package *ape* to extract the coalescent trees generated by *ms*, and analyze the distribution of their external branch lengths (Paradis et al., 2004). We used the external branch length distribution to build a theoretical prediction for the distribution of singletons from each tree (see section 2), and summarized the theoretical distributions by computing mean values and standard errors for each subsample. The *L* coalescent simulations were replicated 200 times.

### 3.2. Range expansions in Africa

Simulations of range expansions were performed by using the computer program SPLATCHE2 based on an array of 87 by 83 demes modeling the African continent (Currat et al., 2004). The demographic scenarios corresponded to range expansions from a single origin, simulated for a total duration of 1, 600 generations. For each deme, the migration rate was equal to *m* = 0.07, and the growth rate was equal to *r* = 0.1. Additional parameters included an ancestral effective population size of 200 individuals, 200 generations before onset of expansion, and an effective mutation rate of 10^−5^ per base pair per generation.

Four types of demographic scenarios were considered. Two scenarios considered a “homogeneous” environment, for which the deme carrying capacities were set to a constant value *C* = 100 everywhere in Africa. Two other scenarios considered a heterogeneous environment linked to vegetation. In tropical semi-desert areas, the carrying capacities were set to *C* = 60, and in tropical extreme deserts and rain forests, the carrying capacities were set to *C* = 30. Demographic histories also differed by their geographic source of expansion. Range expansions were started either from an origin in West Africa (Mali, −4° E, 13° N) or from an origin in the Sahel area (Chad, 22° E, 20° N).

Ten haploid chromosomes were simulated for 30 population samples through the geographic range considered (300 chromosomes). Genetic variation was surveyed at 30,000 loci, and filtered out for monomorphic loci. From the resulting data sets, we computed the empirical distribution of singletons in each population sample, and compared this measure to expected heterozygosity for each population sample. Data files for running the SPLATCHE2 simulations are provided in Supplementary File 1. We reproduced the four scenarios by using individual sampling instead of population sampling. Here, individual genotypes were recorded at 300 distinct geographic sites, each obtained from a Gaussian perturbation of population centers with standard error of 2°. The Kriging method was used to interpolate the values of the expected heterozygosity and the empirical distribution of singletons on a geographic map of Africa (Cressie, 2015).

### 3.3. Pearl millet data

Whole genome sequencing data were obtained for 146 cultivated accessions of pearl millet (*Pennisetum glaucum [L.] R. Br*.) from the species range in Africa (International Pearl Millet Genome Sequencing Consortium, Varshney et al., 2017). A total of 169,095 SNPs were sampled after filtering out low quality variants, and were used to estimate the distribution of singletons (Supplementary Material 1).

### 3.4. Approximate bayesian computation

We used Approximate Bayesian Computation (ABC) to evaluate the ability of the distribution of singletons to correctly estimate the onset of expansion in a range expanding species, and to estimate a posterior distribution for the location of this origin for cultivated pearl millet. We performed 20,000 range expansion simulations by considering a heterogeneous environment using the computer program SPLATCHE2. The deme carrying capacities were equal to *C* = 100 for tropical semi-desert areas, *C* = 20 for tropical extreme deserts and *C* = 10 for rain forests. Additional parameters included an ancestral effective population size of 200 individuals, 200 generations before onset of expansion, and an effective mutation rate of 10^−5^ per base pair per generation.

Prior distributions allowed the geographic coordinates of the origin of expansion to vary over the Sahel region. Longitude ranged between −16°E and 40°E, and latitude ranged between 5°N and 30°N. Lower prior probabilities were given to extreme latitudes and longitudes as a consequence of unsuitable habitats (water regions). Uninformative prior distributions were considered for the migration rate, the growth rate, the total duration of the demographic phase, the ancestral population size and the time before onset of expansion (Supplementary Table 1). In simulations, genetic variation was surveyed at 146 geographic sites corresponding to the exact sampling locations of pearl millet accessions. Ten thousands SNPs were simulated for each genotype. When evaluating summary statistics, a fraction of SNPs were removed from the simulated data in order to match with the amount of missing values observed in the original data set.

**Figure 1:**
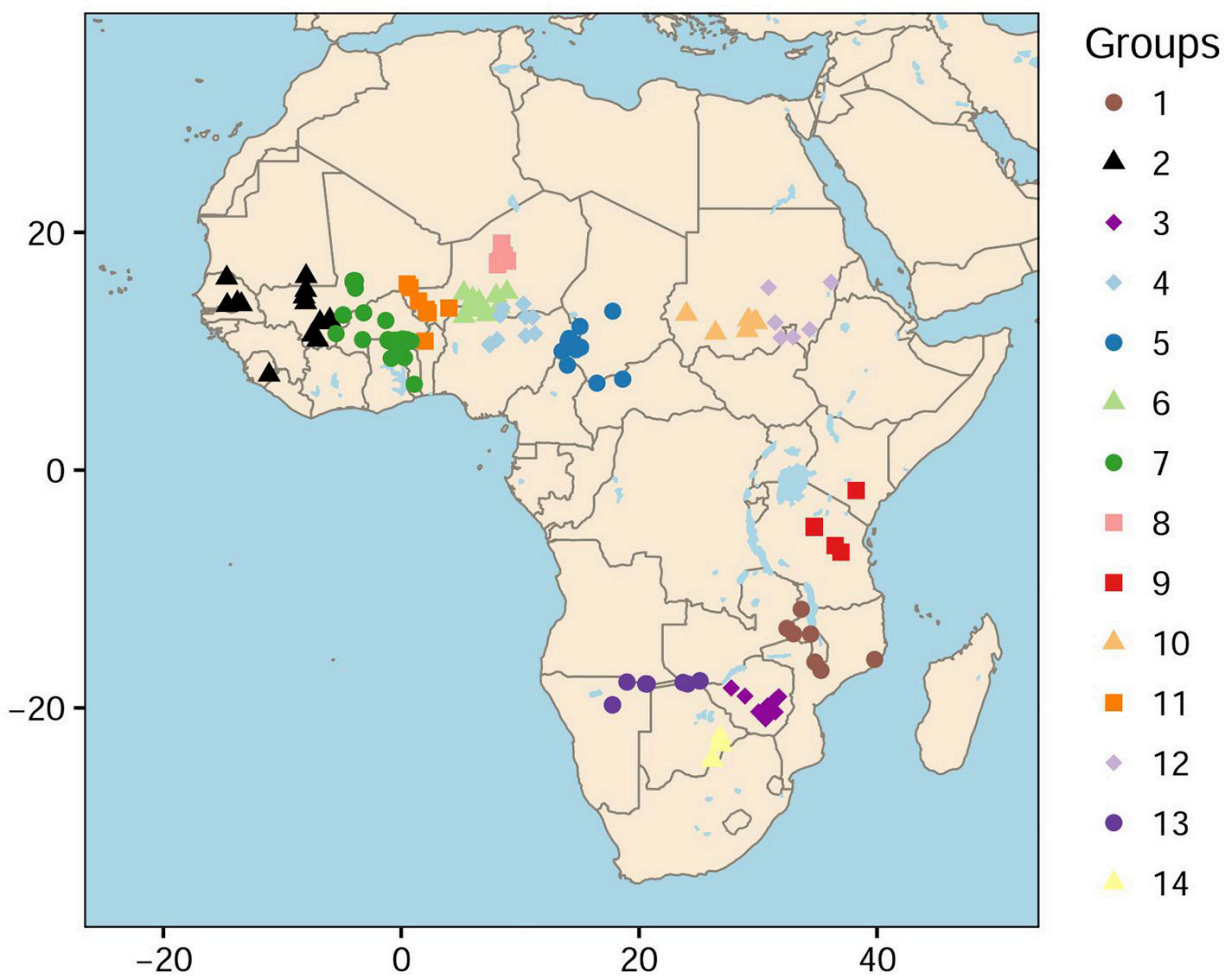
Geographic distribution of 146 cultivated accessions of pearl millet. Fourteen geographic classes were defined as a result of a *k*-means procedure.

To define the summary statistics for ABC, we used a histogram for the distribution of singletons in the sample. The 146 accessions were grouped into spatial clusters according to a *k*-means algorithm and individual geographic information (Hartigan and Wong, 1979). The *k*-means algorithm resulted in 14 groups with more than 6 accessions in each group (Figure 1). To obtain a histogram, we computed the mean number of singletons in each group, and divided this value by the total number of singletons in the sample (Supplementary Table 2). Then ABC analysis was performed with the R package abc (Blum and François, 2010; Csilléry et al., 2012). Neural network models were used to estimate posterior distributions for the latitude and longitude of the geographic onset of expansion whereas the other parameters were considered as nuisance parameters without any interpretable unit. The tolerance rate was set to 0.05 and 250 neural networks were used in the abc function.

We first tested the accuracy of our estimates by using simulated data sets as inputs to the inference method. The sampling procedure and the ABC estimation were replicated 100 times, and we evaluated the correlation between coordinates of true origins and their estimated values. Then we considered the pearl millet data, and represented the prior and posterior densities of the geographic onset parameters by using two-dimensional kernel density estimation with 100 grid points in each direction.

## 4. Results

### 4.1. Coalescent simulations of splitting populations

To evaluate statistical bias in the estimation of the distribution of singletons, we performed coalescent simulations of samples from two populations with unequal genetic diversity. The two populations diverged from an ancestral population *t* generations ago (*split time*), and at split time, the size of population 2 shrinked to *s* times the size of population 1 (*shrink rate*).

**Figure 2:**
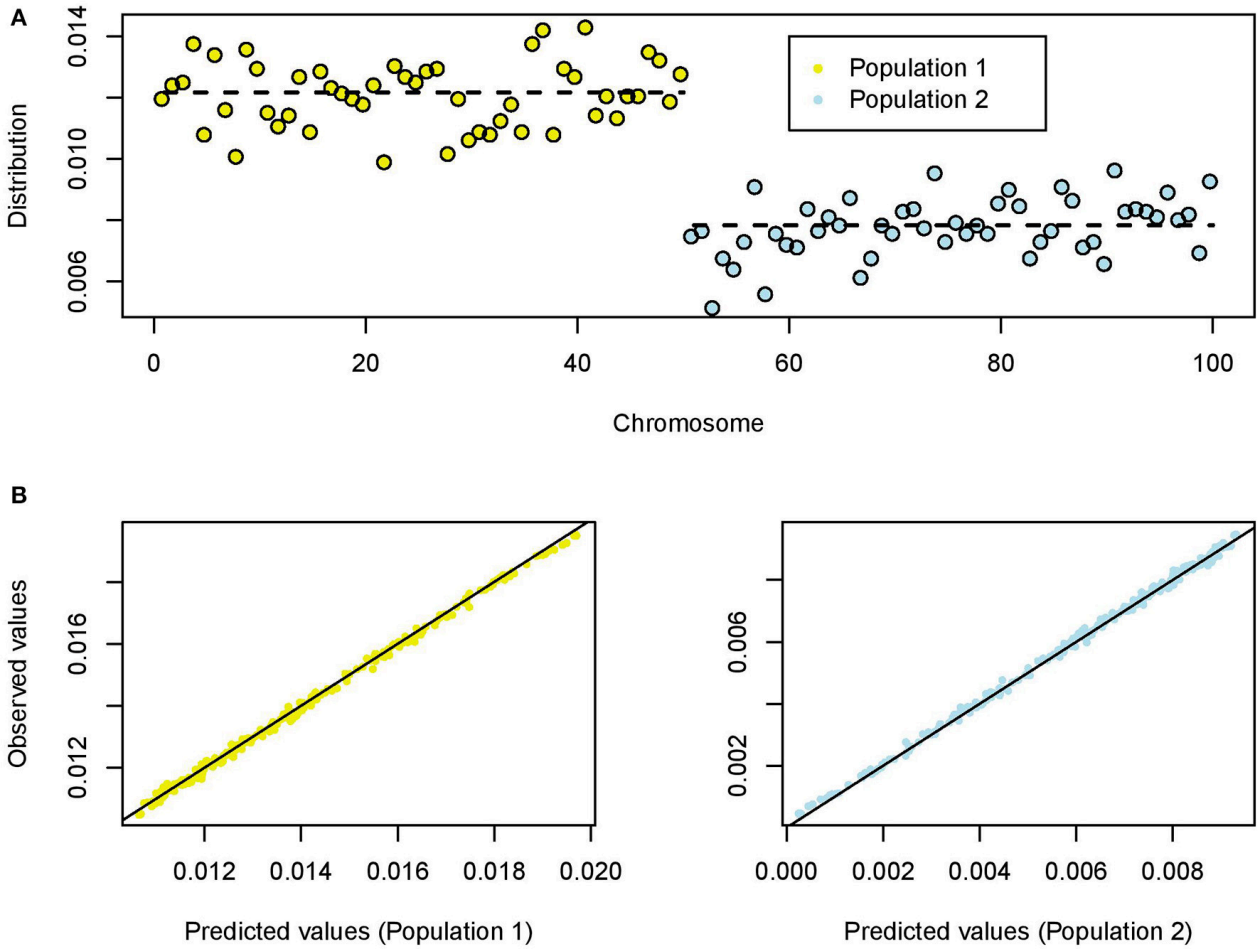
Coalescent simulations of two splitting populations (100 chromosomes). **(A)** Empirical distribution of singletons for a value of the shrink rate *s* = 0.33. The dashed lines represent the averaged values for population 1 (expanding) and population 2 (skrinking). **(B)** Predicted and observed (empirical) values of the distribution of singletons for population 1 (left) and population 2 (right).

For each simulation, the number of polymorphic loci ranged between 7,883 and 39,761 (average value: 25,265 loci). For a value of the shrink rate *s*≈1/3, the average proportion of singletons in population 1 was about π_1_ = 0.0122, and the average proportion of singletons in population 2 was about π_2_ = 0.0078 (π_1_+π_2_ = 2/*n*). This result reflected that genetic diversity in population 1 was higher than in population 2. The ratio was about π_1_/π_2_ = 1.55 (Figure 2A). The individual proportions were concentrated around their mean values with relatively small standard deviations (SD_1_ = 0.0010, SD_2_ = 0.0008).

The results from 200 replicates provided clear evidence that the empirical distribution of singletons is an unbiased estimate of its theoretical distribution based on coalescent trees (Figure 2B). The split time parameter had a weak influence on the distribution of singletons (Pearson correlation test, *P* = 0.64). The ratio π_1_/π_2_ reached values between 10 and 40 when the shrink rate was below 10%, and this parameter had a strong influence on the empirical distribution of singletons (Figure S1).

### 4.2. Range expansions in Africa

**Figure 3:**
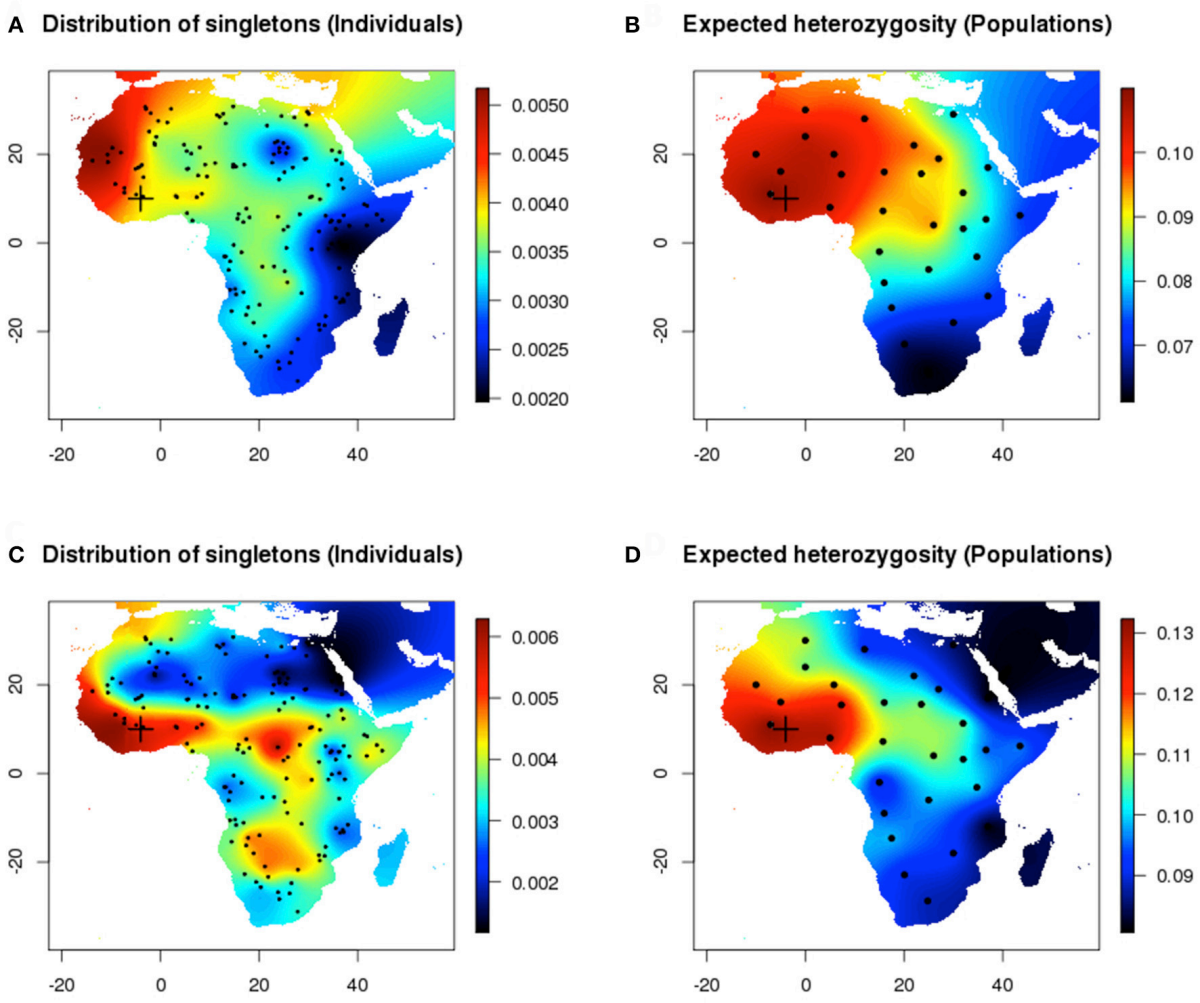
Individual vs. population sampling after a range expansion simulation scenario (Western origin). **(A,B)** Homogeneous environment. Maps of the empirical distribution of singletons (individual sampling) and expected heterozygosity (true population sampling). **(C,D)** Inhomogeneous environment.

For data sets generated under range expansion scenarios, the number of polymorphic loci ranged between 25,453 and 29,321 loci. The number of singletons ranged between 8,835 and 12,653, and the site frequency spectrum showed an excess of rare alleles as expected under explosive population growth. When the onset of expansion was set in Western Africa (cross in Figure 3), the maps of the empirical distribution of singletons and expected heterozygosity exhibited similar large-scale geographic patterns (Figure 3, Pearson's correlation coefficient 0.78). Because the computation of expected heterozygosities was based on a perfect assignment of samples to their true populations of origin, the interpolated maps corresponding to this measure (Figures 3B,D) contained less uncertainty than the maps of singletons (Figures 3A,C) that were based on random individual sampling. Considering environmental heterogeneity increased the variability of spatial estimates (Figures 3C,D).

**Figure 4:**
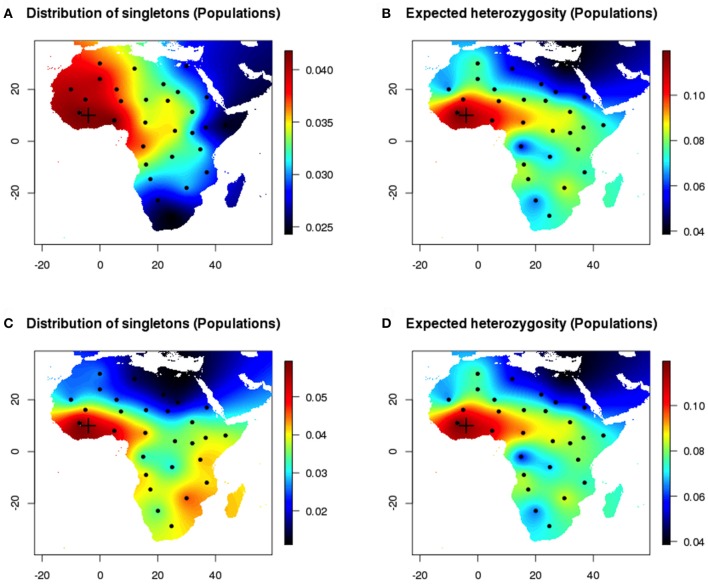
Population sampling after a range expansion simulation scenario (Western origin). **(A,B)** Homogeneous environment. Maps of the empirical distribution of singletons (true population sampling) and expected heterozygosity (true population sampling). **(C,D)** Inhomogeneous environment.

Next, we compared estimates of heterozygosity for populations to the distribution of singletons in the same populations (Figure 4). Differences between maps produced with the empirical distribution of singletons and with expected heterozygosity decreased when the sampled chromosomes were perfectly assigned to their population of origin. The individual and population-based measures provided concordant estimates of genetic diversity in geographic space (Pearson's correlation coefficient 0.51). Similar results were observed when the onset of expansion was set in the Sahel area (20° E, 22° N) and were reported in Figures S2, S3.

### 4.3. Estimates of expansion onsets and application to pearl millet

**Figure 5:**
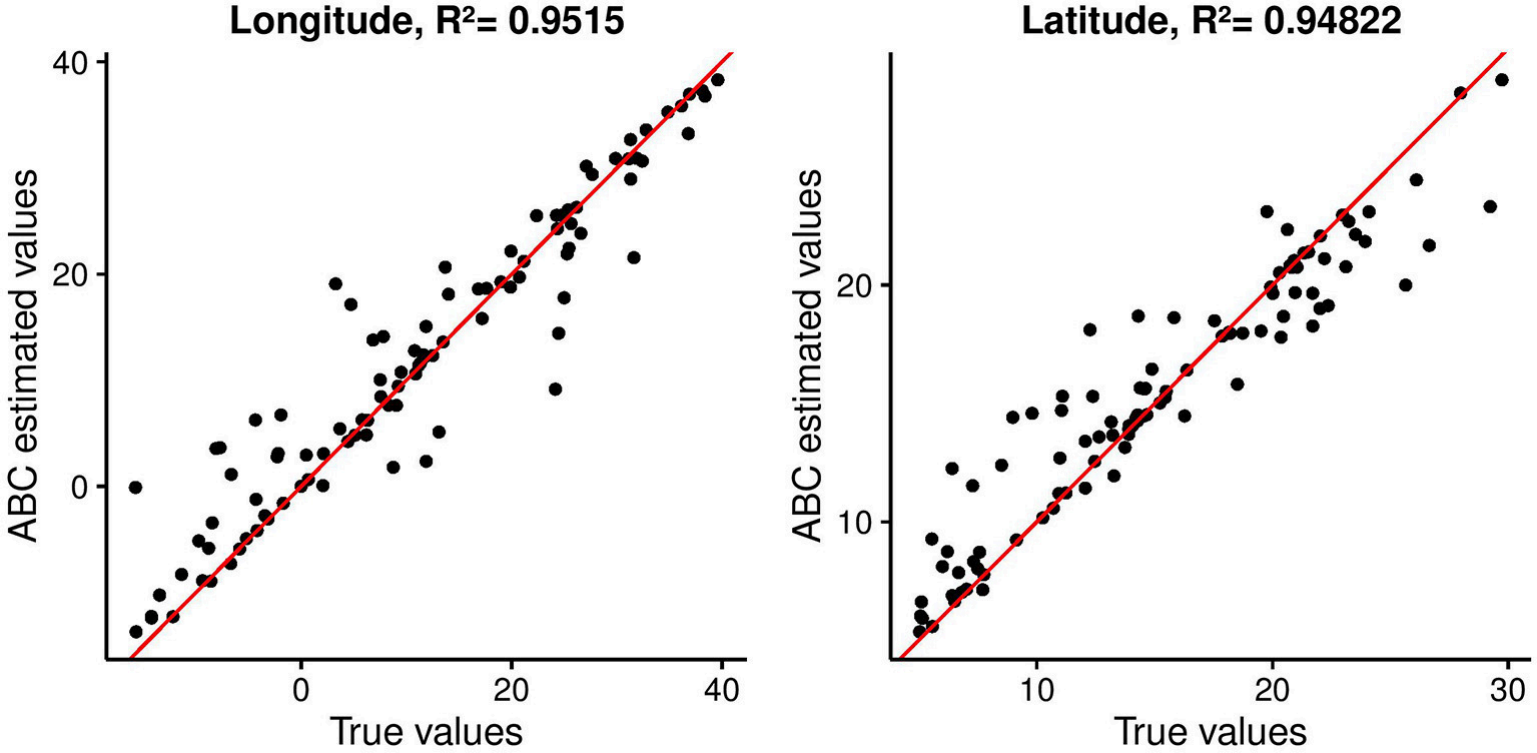
Estimated coordinates of origin against their true values for 100 simulated data sets used as targets for ABC analysis. Pearson's correlation coefficients are reported.

First, we used the distribution of singletons in ABC to infer origins of range expansion in 100 simulated data sets (Figure 5). The results provided evidence of the usefulness of the statistics to identify origins of range expansions. Estimated values for the longitude and latitude of the onset of expansion were highly correlated to the true values for these parameters. Pearson's squared correlation coefficients were equal to *R*^2^ = 0.950 for the longitude and *R*^2^ = 0.948 for the latitude (*p*-values < 0.01).

**Figure 6:**
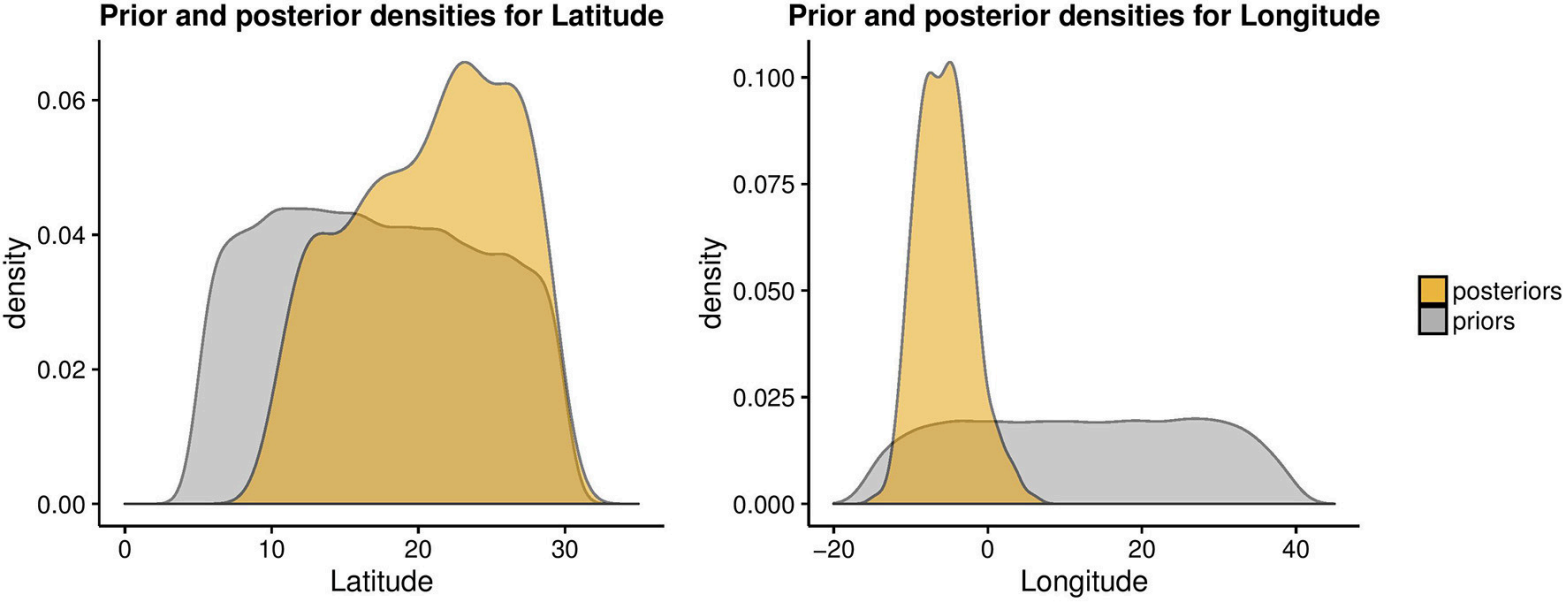
Prior and posterior density estimates for the longitude and latitude of the expansion onset for cultivated pearl millet in Africa.

**Figure 7:**
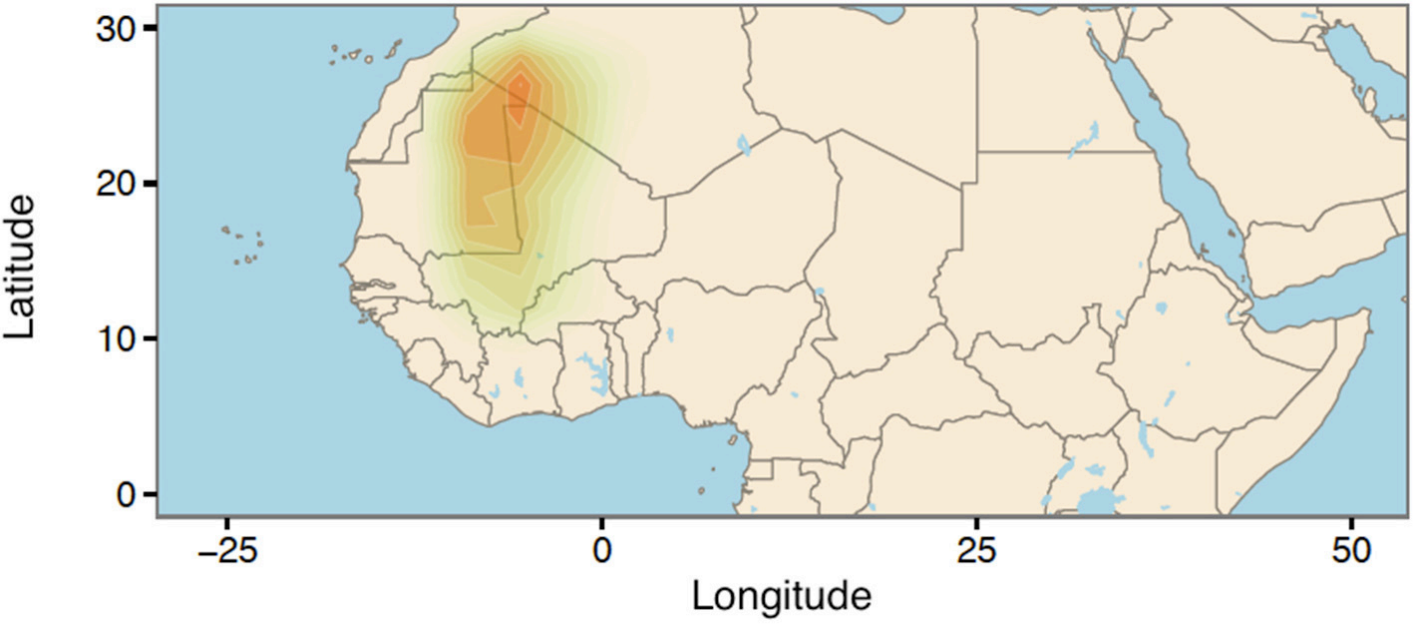
Geographic origin of cultivated pearl millet expansion using kernel density estimation.

Next, we used the ABC approach to provide insights on the origin of range expansion of cultivated pearl millet in Africa. A total number of 41,032 singletons were found for 146 individuals, representing 24.27% of all variants. The posterior density for the longitude exhibited a mode around −7.52°E (CI:-11.26°E, 0.84°E) (Figure 6). For the latitude of origin, the posterior density exhibited a mode around 24.2°N and a large credible interval (CI: 11.03°N, 29.06°N) (Figure 6). The most probable location for the origin of expansion of pearl millet in Africa was found near the Mali-Mauritania border (Figure 7).

## 5. Discussion

How singletons are distributed across geographic space provides a local measure of genetic diversity that can be measured at the individual level. In this study, we developed a theoretical background for the empirical distribution of singletons in a sample of chromosomes. We used simulations to provide evidence that the empirical distribution of singletons measures individual contributions to genetic diversity in the sample. The main advantage of this approach is to provide individual-based (local) estimates of genetic diversity that do not require the definition of populations.

Incorporated in an ABC framework, the empirical distribution of singletons led to accurate estimates of the geographic origin of range expansions in simulations. In ABC, the distribution of singletons was estimated by histograms obtained from clustering algorithms, and the histograms were used as summary statistics for Bayesian inference. Those statistics are appropriate to analyze the results of sequencing projects based on large scale sampling of individuals across geographic space. The method can be viewed as an interesting alternative to phylogenetic approaches when genomic sequences are used.

Potential factors that could bias our estimates of local genetic diversity includes missing data, genotyping errors, related individuals, and the use of a folded site frequency spectrum. Missing values or genotyping errors impacts individual data regardless of geography. By sharing genomic variation locally, related individuals reduce the number of unique variants drastically, and generate bias in global estimates of genetic diversity. Though those errors increase uncertainty in estimates, the biases on geographic estimates remain at small levels. Our ABC analysis took the potential biases into account by simulating the missing data, genotyping errors and the other issues. Alternative methods that could remove the biases would be based on genotype imputation and on the availability of genomic data from a closely related species.

We provided an illustration of the potential of singletons to inform demographic history by studying range expansion of pearl millet in Africa. Pearl millet is a widely grown staple crop in Africa and India, but its precise origin is currently unknown (Tostain, 1992; Oumar et al., 2008; Clotault et al., 2012). When we applied an ABC approach to cultivated pearl millet genomes, we obtained a result supporting the Northern Mali region as the most probable geographic origin of expansion. Although the accuracy of the ABC approach was validated with extensive computer simulations of range expansion, the empirical results pointed out some limitations of our model for the data. The uncertainty around 18° reported for the latitude of origin was high, and improving our estimate would require supplementary information on past environmental conditions, carrying capacities and gene flow between pearl millet and related species. Interestingly, our results rejected an eastern origin for the expansion of the domesticated cereal. This result is consistent with recent archeological studies using both wild and cultivated samples, that pinpointed the Mali-Niger region as the most likely origin of domestication of pearl millet (Manning et al., 2011; Ozainne et al., 2014).

To conclude, singletons are a major component of the site frequency spectrum for many model and non-model species. The density of singletons in genomes has recently proven useful to detect selection in human genomes (Field et al., 2016). Here we showed that the density of singletons in geographic space is useful for providing local estimates of genetic diversity and key insights on the demographic history of a species.

## Author contributions

All authors listed, have made substantial, direct and intellectual contribution to the work, and approved it for publication.

### Conflict of interest statement

The authors declare that the research was conducted in the absence of any commercial or financial relationships that could be construed as a potential conflict of interest.

## Supplementary material

Figure S1 Averaged proportion of singletons in population 1, and standard deviations in populations 1 and 2, as functions of the shrink rate.

Figure S2 Individual vs. population sampling after a range expansion simulation scenario (Sahel origin). **(A,B)** Homogeneous environment. Maps of the empirical distribution of singletons (individual sampling) and expected heterozygosity (true population sampling). **(C,D)** Inhomogeneous environment.

Figure S3 Population sampling after a range expansion simulation scenario (Sahel origin). **(A,B)** Homogeneous environment. Maps of the empirical distribution of singletons (true population sampling) and expected heterozygosity (true population sampling). **(C,D)** Inhomogeneous environment.
