# Supplementary figures and images for: The Empirical Distribution of Singletons for Geographic Samples of DNA Sequences
Source: Front Genet. 2017 Sep 29;8:139. doi: 10.3389/fgene.2017.00139 (PMC5627571; doi:10.3389/fgene.2017.00139)

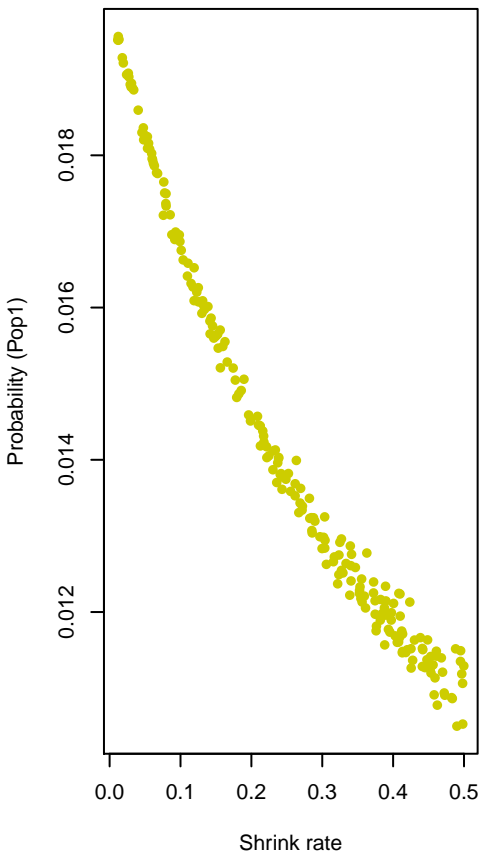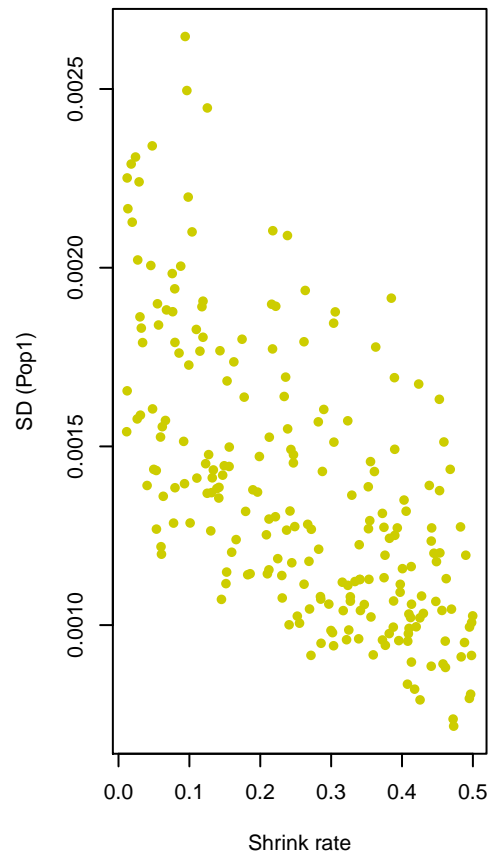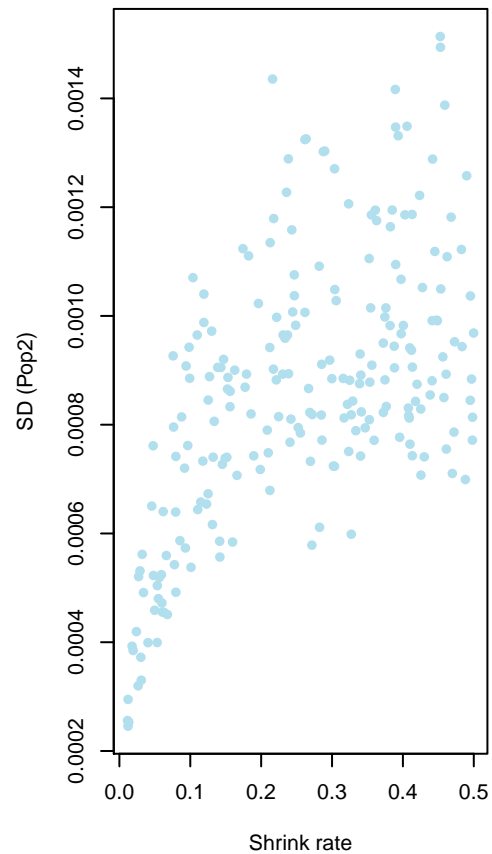

Supplement: Figure S1 — Averaged proportion of singletons in population 1, and standard deviations in populations 1 and 2, as functions of the shrink rate. [file Image1.PDF]

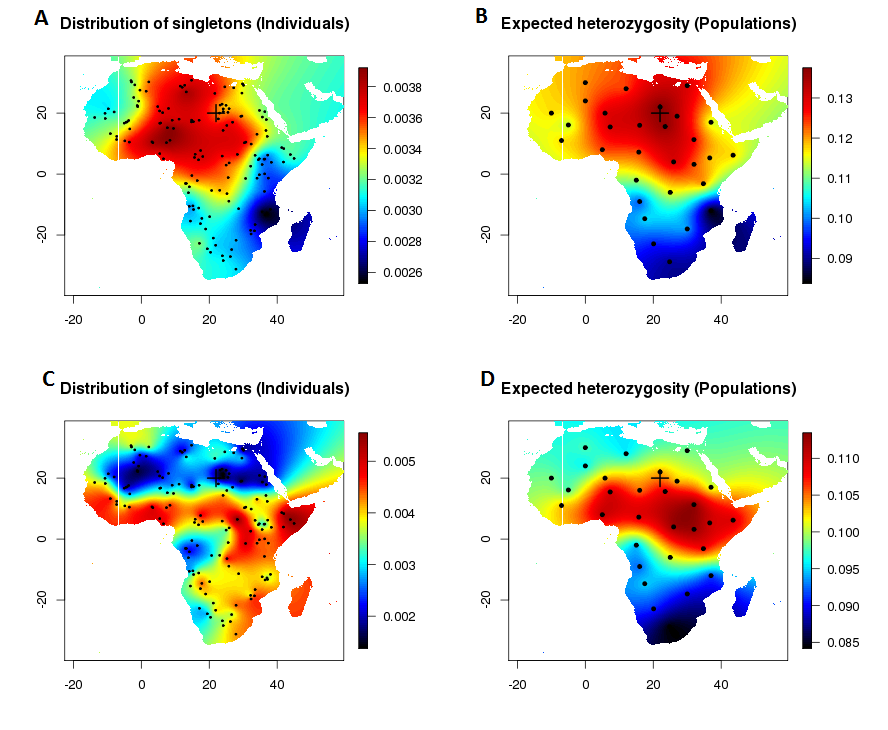

Supplement: Figure S2 — Individual vs. population sampling after a range expansion simulation scenario (Sahel origin). (A,B) Homogeneous environment. Maps of the empirical distribution of singletons (individual sampling) and expected heterozygosity (true population sampling). (C,D) Inhomogeneous environment. [file Image2.PNG]

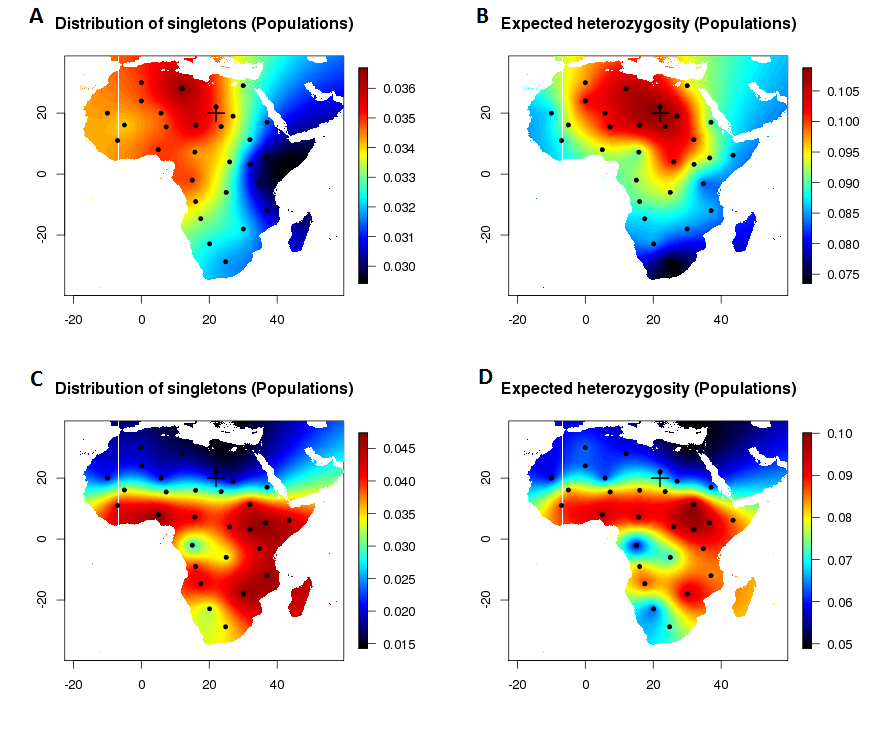

Supplement: Figure S3 — Population sampling after a range expansion simulation scenario (Sahel origin). (A,B) Homogeneous environment. Maps of the empirical distribution of singletons (true population sampling) and expected heterozygosity (true population sampling). (C,D) Inhomogeneous environment. [file Image3.PNG]
