# Supplementary material for: The Empirical Distribution of Singletons for Geographic Samples of DNA Sequences
Source: Front Genet. 2017 Sep 29;8:139. doi: 10.3389/fgene.2017.00139 (PMC5627571; doi:10.3389/fgene.2017.00139)
Supplement: Supplementary file 5 [file Table1.DOCX]

**Supplementary Table 1**. Prior distributions for spatial coalescent ABC simulations

| Parameter | Prior distribution | Minimum (range) | maximum (range) |
| --- | --- | --- | --- |
| Expansion  rate | uniform | 0.05 | 0.9 |
| Migration  rate | uniform | 0.03 | 0.5 |
| Total  duration | uniform | 1500 | 400 |
| Ancestral  population size | uniform | 200 | 1000 |
| Time before onset  of expansion | uniform | 200 | 1000 |
| Latitude  (°E) | uniform | -16 | 40 |
| Longitude  (°N) | uniform | 5 | 30 |
