# Supplementary material for: The Empirical Distribution of Singletons for Geographic Samples of DNA Sequences
Source: Front Genet. 2017 Sep 29;8:139. doi: 10.3389/fgene.2017.00139 (PMC5627571; doi:10.3389/fgene.2017.00139)
Supplement: Supplementary file 6 [file Table2.DOCX]

**Supplementary Table 2**. Summary statistics used in an ABC analysis of the pearl millet data.

| Group (k-means) | Density of singletons (%) |
| --- | --- |
| 1 | 6.37 |
| 2 | 9.43 |
| 3 | 6.13 |
| 4 | 6.95 |
| 5 | 7.39 |
| 6 | 6.51 |
| 7 | 7.39 |
| 8 | 6.67 |
| 9 | 5.34 |
| 10 | 11.08 |
| 11 | 7.23 |
| 12 | 8.50 |
| 13 | 6.03 |
| 14 | 5.01 |
