# Supplementary material for: The Empirical Distribution of Singletons for Geographic Samples of DNA Sequences
Source: Front Genet. 2017 Sep 29;8:139. doi: 10.3389/fgene.2017.00139 (PMC5627571; doi:10.3389/fgene.2017.00139)
Supplement: Supplementary file 7 [file Table3.DOCX]

## Supplementary File 1 : SPLATCHE Input file for one spatial simulation (homogeneous environment)

## SPLATCHE setting file :

PopDensityFile=./datasets_1layer/dens_init.txt

PresVegetationFile=./datasets_1layer/ppveg.asc

RoughnessTopoFile=./datasets_1layer/roughness.asc

Veg2KFile=./datasets_1layer/dynamic_K.txt

Veg2FFile=./datasets_1layer/dynamic_F.txt

ChosenDemographicModel=3

EndTime=1600

GenerationTime=1

GrowthRate=0.1

MigrationRate=0.07

AllowSourcePopulationOverflow=1

TauValue=200

AncestralSize=200

ArrivalCellFile=./datasets_1layer/Arrival_cell.col

FrictionChoice=2

RealBPTime=-120000

RiverFrictionChangeFactor=0.5

RiverCarCapChangeFactor=2

CoastFrictionChangeFactor=0.5

CoastCarCapChangeFactor=2

SampleFile=./datasets_1layer/GeneSamples.sam

GeneticFile=./datasets_1layer/genetic_data_SEQ.par

NumGeneticSimulations=60

GenotypicData=1

MaxNumGenerations=10000

NexusFile=0

GenealogiesFile=0

ImmigDistrFile=0

DoublePopulationMode=0

ChosenDemographicModel_P2=22

GrowthRate_P2=0.4

MigrationRate_P2=0.4

MigrRate_P1_to_P2=0.0

MigrRate_P2_to_P1=0.0

Veg2K_P2_File=./datasets_1layer/veg2K_neol.txt

Veg2F_P2_File=./datasets_1layer/veg2F_0.txt

Veg2m_P2_File=./datasets_2layers/dynamic_m_P2.txt

PropFile=1

GenerateOutputMigrationBMP=0

GenerateOutputMDensityBMP=0

GenerateOutputOccupationBMP=0

GenerateOutputMigrationASCII=0

GenerateOutputMDensityASCII=0

GenerateOutputOccupationASCII=0

AllowShortIntForNumberOfIndividuals=1

## Initial density file

1

source1 200 13 -4 0 0 0 0 0 0

Pop_name pop_size Lat Long ResizeA Time_ResizeB ResizeB MigrationFromCurrentSource NoLayer TimeOfExpansion

## DynamicK

1

1 ./datasets_1layer/veg2K_pop1_time_1.txt Time1

## Dynamic_F

1

1 ./datasets_1layer/veg2F_pop1_time_1.txt Time1

## veg2F_pop1_time_1.txt

1 0.5 Tropical rainforest

2 0.5 Monsoon or dry forest

3 0.5 Tropical woodland

4 0.5 Tropical thorn scrub and scrub woodland

5 0.5 Tropical semi-desert

6 0.5 Tropical grassland

7 0.5 Tropical extreme desert

8 0.5 Savanna

9 0.5 Broadleaved temperate evergreen forest

10 0.5 Montane tropical forest

11 0.5 Mediterranean sclerophyll woodland or forest

12 0.5 Temperate deciduous broadleaved forest

13 0.5 Southern taiga

14 0.5 Mid Taiga

15 0.5 Open boreal woodlands

16 0.5 Semi-arid temperate woodland or scrub

17 0.5 Semi aride temperate scrub

18 0.5 Tundra

19 0.5 Steppe-tundra

20 0.5 Polar and alpine desert

21 0.5 Temperate desert

22 0.5 Temperate semi-desert

23 0.5 Forest steppe

24 0.5 Forest tundra

25 0.5 Montane Mosaic

26 0.5 Dry Steppe

27 0.5 Temperate Steppe Grassland

28 1 Bog/swamp

29 1 Ice sheet and other permanent ice

30 1 Lakes and open water

31 0.5 Ponts (japon, Sardaigne, orcadian, australie)

## veg2K_pop1_time_1.txt

1 100 Tropical rainforest

2 100 Monsoon or dry forest

3 100 Tropical woodland

4 100 Tropical thorn scrub and scrub woodland

5 100 Tropical semi-desert

6 100 Tropical grassland

7 100 Tropical extreme desert

8 100 Savanna

9 100 Broadleaved temperate evergreen forest

10 100 Montane tropical forest

11 100 Mediterranean sclerophyll woodland or forest

12 100 Temperate deciduous broadleaved forest

13 100 Southern taiga

14 100 Mid Taiga

15 100 Open boreal woodlands

16 100 Semi-arid temperate woodland or scrub

17 100 Semi aride temperate scrub

18 100 Tundra

19 100 Steppe-tundra

20 100 Polar and alpine desert

21 100 Temperate desert

22 100 Temperate semi-desert

23 100 Forest steppe

24 100 Forest tundra

25 100 Montane Mosaic

26 100 Dry Steppe

27 100 Temperate Steppe Grassland

28 0 Bog/swamp

29 0 Ice sheet and other permanent ice

30 0 Lakes and open water

31 100 Ponts (japon, Sardaigne, orcadian, australie)

## GeneSamples.sam

150

32 2 0 17.5756133465451 -3.73792435381425

32 2 0 16.7152091157424 -5.03292948655203

32 2 0 17.027845424196 -4.48386965795806

32 2 0 14.8055517489053 -3.39357018345316

32 2 0 17.093876973644 -4.21519790942834

98 2 0 17.0108068514248 26.8575335987809

98 2 0 20.2437075698365 25.4994585531386

98 2 0 18.0912415078504 22.6469146012224

98 2 0 14.3295957780227 24.066915831385

98 2 0 15.9468980654113 25.1679540214167

83 2 0 13.2660518486305 -9.11489455272764

83 2 0 12.3762349975459 -7.23959436598338

83 2 0 10.8836287883418 -4.85410193706182

83 2 0 10.4896886815939 -3.55307552475877

83 2 0 11.3332095615163 -7.27649945103816

21 2 0 3.94791434625338 41.8286679196994

21 2 0 8.35579988470627 41.9434624232274

21 2 0 6.39848445098147 43.8819993467891

21 2 0 8.68750205092289 41.3396808585156

21 2 0 5.13486803924117 44.9787878180085

64 2 0 17.6734977213203 9.0736202861866

64 2 0 16.7634157396181 5.66608735721736

64 2 0 15.1894656627649 7.65159270058272

64 2 0 14.9722127629744 9.47156013047215

64 2 0 11.0980935046915 8.43719937756437

87 2 0 5.03764032363667 34.7202025850058

87 2 0 4.70420177895359 34.4518188382805

87 2 0 -0.223553268464056 34.7408176026296

87 2 0 2.59110624724152 32.1824773239347

87 2 0 -1.44110296700322 30.6951487127121

55 2 0 4.67730676119389 16.8312617911307

55 2 0 5.75896353368946 18.091869414583

55 2 0 7.72930747957032 17.4489273305954

55 2 0 5.45108381121152 15.6562960112589

55 2 0 6.51139774149578 15.8470037056219

76 2 0 -2.13553070091179 16.4590050174196

76 2 0 -0.489077502288203 14.8833501149492

76 2 0 -3.06999640820746 12.9918575081187

76 2 0 -4.18236496033204 14.1325489990238

76 2 0 -3.07496070899961 13.2437553594378

17 2 0 13.0737656974688 34.0499807403332

17 2 0 12.3619627915423 37.069493688837

17 2 0 10.4943070291296 30.7716503135921

17 2 0 9.85029042436972 32.3279789012576

17 2 0 10.2691577997732 30.344909383691

78 2 0 -6.12176066838343 13.8515936050202

78 2 0 -10.566238044815 14.1093299757618

78 2 0 -10.4299936027296 15.1853063685448

78 2 0 -11.1343779845681 16.896430124544

78 2 0 -11.6276775022307 15.3731163590819

11 2 0 6.21759672391043 37.1997729968518

11 2 0 4.92479508121537 36.3637704734

11 2 0 3.86567195758512 38.2800666780029

11 2 0 6.16068361365958 33.4975142815521

11 2 0 4.8957137526657 36.1322089776412

43 2 0 21.3800488379037 24.0964306543842

43 2 0 18.944182490158 29.2978664546145

43 2 0 19.0436720818216 29.6101467911404

43 2 0 22.9552872868454 23.7738360129409

43 2 0 21.4482906473044 25.8364940138684

12 2 0 -1.34969055418583 34.065054124993

12 2 0 3.12928229023952 35.5658906011082

12 2 0 -5.68562552057254 36.2517842837924

12 2 0 -1.32015415789665 37.3785094783631

12 2 0 0.0723808953790739 36.3064612324216

23 2 0 22.1393147565188 4.82878232571732

23 2 0 20.7033277389223 5.36662666561094

23 2 0 20.1408345701298 6.27523215408667

23 2 0 21.5341443795146 8.00194828236627

23 2 0 16.6752638802183 5.42609141143326

13 2 0 -18.023609672204 18.9341782689534

13 2 0 -16.258614055357 17.3973488687936

13 2 0 -13.9714521695506 20.1141904851058

13 2 0 -14.5298361922345 17.8207362723308

13 2 0 -15.4057144674844 14.4206217033699

98 2 0 -13.2279130537647 37.1560357131009

98 2 0 -12.3784140445439 35.5686286942869

98 2 0 -12.7382106277324 36.9873083231787

98 2 0 -11.5776803651829 38.0212955215525

98 2 0 -13.4080022756832 36.4296592938011

14 2 0 -25.6544037579256 20.374474211525

14 2 0 -20.992307608666 21.1700727786658

14 2 0 -24.5138381754854 19.4131573568172

14 2 0 -22.7163443194094 17.0954766216171

14 2 0 -23.3448013642826 21.8810739362928

9 2 0 -16.5679454116815 33.500211186705

9 2 0 -18.6194249619159 33.2979575218543

9 2 0 -18.4592683772184 32.1731865030717

9 2 0 -19.6572444919871 32.3014361217718

9 2 0 -21.6971479539387 27.8888208076836

16 2 0 -31.176174798208 27.8546247795419

16 2 0 -24.7650152595392 26.5895144225329

16 2 0 -27.0703720155876 26.2150803744623

16 2 0 -28.3739205181763 24.1112148929973

16 2 0 -26.8112699648411 23.8530411313967

99 2 0 -5.38109819355612 20.6946133530189

99 2 0 -5.32443155715229 23.0758935331344

99 2 0 -8.88836637688127 25.7727328251871

99 2 0 -6.42110521747852 25.2148605952962

99 2 0 -11.3572260933807 28.6427428083997

51 2 0 5.90800386737316 23.8059798801663

51 2 0 6.45486712394841 28.2705181954742

51 2 0 -1.15824532376345 25.5658968644953

51 2 0 3.65690502142008 25.8389655683588

51 2 0 2.94078137168683 24.9601220136273

52 2 0 20.3738106303714 -8.01352891686373

52 2 0 18.5612654089119 -13.7368442177905

52 2 0 21.4112766751261 -9.73483840122908

52 2 0 19.9390260865685 -10.692112236575

52 2 0 18.2312498288208 -10.6238345149001

53 2 0 22.1131619018682 -1.07141234405085

53 2 0 27.1849630305841 1.41457482596915

53 2 0 23.9291788357312 -0.800114756165911

53 2 0 22.4870956864226 -1.2102121232934

53 2 0 25.1235676211707 -2.24068602383239

65 2 0 17.1937730083609 15.3217297523352

65 2 0 17.1007242508151 14.7672686174126

65 2 0 17.6702065161985 17.008499800636

65 2 0 18.0245653280222 11.1064734760015

65 2 0 17.7048849972173 14.8102817373335

56 2 0 19.9995287347878 23.0039056790762

56 2 0 20.4868344887437 24.1026770284575

56 2 0 26.4655472721309 21.6436580941138

56 2 0 22.5665636952934 24.9387767320733

56 2 0 22.6281048911978 23.2008702346664

57 2 0 17.863122143812 36.8023070544765

57 2 0 20.4709186282733 36.3623371095382

57 2 0 14.3027064566475 36.6930473628405

57 2 0 18.7076680143677 35.6183807486882

57 2 0 20.8794302040778 34.7804354162723

58 2 0 30.2284058618551 2.21015534949605

58 2 0 30.6525746386815 -3.03516652815139

58 2 0 29.2519042444928 -1.91941723237457

58 2 0 27.5349292128729 -0.40741527736942

58 2 0 30.2004987802408 -2.91445899078414

59 2 0 29.1403139098952 12.7708154569821

59 2 0 30.7789093507968 14.7776399370873

59 2 0 28.4694938123239 12.026406685163

59 2 0 26.9424075443157 13.2131350509032

59 2 0 28.8602653148848 11.455366814028

60 2 0 28.2945026687108 27.1956296041748

60 2 0 29.1173976601488 30.0876903598043

60 2 0 26.5694067528447 29.6190331705121

60 2 0 28.6017973500437 24.5846717788939

60 2 0 29.595899198718 29.7764824574726

61 2 0 10.2796740521598 3.39780663074004

61 2 0 10.3120242764785 7.39632395247894

61 2 0 10.5663494461623 3.1369272696776

61 2 0 6.70216553596587 5.43192817691947

61 2 0 5.02019217723452 6.50524670803864

## genetic_data_SEQ.par

500 //Num chromosomes

#chromosome 1, //per Block:data type, number of loci, per generation recombination, per site mutation rates and transition rate (fraction of substitutions that are transitions)

1

DNA 1 0 0.000011 1

#chromosome 2, //per Block:data type, number of loci, per generation recombination, per site mutation rates and transition rate (fraction of substitutions that are transitions)

1

DNA 1 0 0.000011 1

…

## ppveg.asc

ncols 83

nrows 87

xllcorner -16.733229949463

yllcorner -35.237187

cellsize 0.83

NODATA_value -9999

-9999 -9999 -9999 -9999 -9999 -9999 -9999 -9999 -9999 -9999 -9999 -9999 -9999 -9999 -9999 -9999 -9999 -9999 -9999 -9999 -9999 -9999 -9999 -9999 -9999 -9999 -9999 -9999 -9999 -9999 -9999 3 -9999 -9999 -9999 -9999 -9999 -9999 -9999 -9999 -9999 -9999 -9999 -9999 -9999 -9999 -9999 -9999 -9999 -9999 -9999 -9999 -9999 -9999 -9999 -9999 -9999 -9999 -9999 -9999 -9999 -9999 -9999 -9999 -9999 -9999 -9999 -9999 -9999 -9999 -9999 -9999 -9999 -9999 -9999 -9999 -9999 -9999 -9999 -9999 -9999 -9999 -9999

-9999 -9999 -9999 -9999 -9999 -9999 -9999 -9999 -9999 -9999 -9999 -9999 -9999 -9999 -9999 -9999 -9999 -9999 -9999 -9999 -9999 3 3 3 3 3 3 3 3 3 3 3 3 -9999 -9999 -9999 -9999 -9999 -9999 -9999 -9999 -9999 -9999 -9999 -9999 -9999 -9999 -9999 -9999 -9999 -9999 -9999 -9999 -9999 -9999 -9999 -9999 -9999 -9999 -9999 -9999 -9999 -9999 -9999 -9999 -9999 -9999 -9999 -9999 -9999 -9999 -9999 -9999 -9999 -9999 -9999 -9999 -9999 -9999 -9999 -9999 -9999 -9999

-9999 -9999 -9999 -9999 -9999 -9999 -9999 -9999 -9999 -9999 -9999 -9999 -9999 3 -9999 -9999 -9999 -9999 -9999 3 3 3 3 3 3 3 9 9 9 9 9 9 9 -9999 -9999 -9999 -9999 -9999 -9999 -9999 -9999 -9999 -9999 -9999 -9999 -9999 -9999 -9999 -9999 -9999 -9999 -9999 -9999 -9999 -9999 -9999 -9999 -9999 -9999 -9999 -9999 -9999 -9999 -9999 -9999 -9999 -9999 -9999 -9999 -9999 -9999 -9999 -9999 -9999 -9999 -9999 -9999 -9999 -9999 -9999 -9999 -9999 -9999

-9999 -9999 -9999 -9999 -9999 -9999 -9999 -9999 -9999 -9999 -9999 -9999 3 3 3 3 3 3 3 3 9 9 9 9 9 9 9 9 9 9 9 9 9 -9999 -9999 -9999 -9999 -9999 -9999 -9999 -9999 -9999 -9999 -9999 -9999 -9999 -9999 -9999 -9999 -9999 -9999 -9999 -9999 -9999 -9999 -9999 -9999 -9999 -9999 -9999 -9999 -9999 -9999 -9999 -9999 -9999 -9999 -9999 -9999 -9999 -9999 -9999 -9999 -9999 -9999 -9999 -9999 -9999 -9999 -9999 -9999 -9999 -9999

-9999 -9999 -9999 -9999 -9999 -9999 -9999 -9999 -9999 -9999 -9999 -9999 9 9 9 9 9 9 9 9 9 9 9 9 9 9 7 7 7 7 9 9 -9999 -9999 -9999 -9999 -9999 -9999 -9999 -9999 -9999 -9999 -9999 -9999 -9999 -9999 -9999 -9999 -9999 -9999 -9999 -9999 -9999 -9999 -9999 -9999 -9999 -9999 -9999 -9999 -9999 -9999 -9999 -9999 -9999 -9999 -9999 -9999 -9999 -9999 -9999 -9999 -9999 -9999 -9999 -9999 -9999 -9999 -9999 -9999 -9999 -9999 -9999

-9999 -9999 -9999 -9999 -9999 -9999 -9999 -9999 -9999 -9999 3 3 9 9 9 9 9 9 9 9 9 9 9 7 7 7 7 7 7 7 7 7 7 9 -9999 -9999 -9999 -9999 -9999 -9999 -9999 -9999 -9999 -9999 -9999 -9999 -9999 -9999 -9999 -9999 -9999 -9999 -9999 -9999 -9999 -9999 -9999 -9999 -9999 -9999 -9999 -9999 -9999 -9999 -9999 -9999 -9999 -9999 -9999 -9999 -9999 -9999 -9999 -9999 -9999 -9999 -9999 -9999 -9999 -9999 -9999 -9999 -9999

-9999 -9999 -9999 -9999 -9999 -9999 -9999 -9999 -9999 9 9 9 9 9 9 9 9 9 9 9 9 7 7 7 7 7 7 7 7 7 7 7 7 7 7 7 7 7 -9999 -9999 -9999 -9999 -9999 -9999 9 9 9 9 -9999 -9999 -9999 -9999 -9999 -9999 -9999 -9999 -9999 -9999 -9999 -9999 -9999 -9999 -9999 -9999 -9999 -9999 -9999 -9999 -9999 -9999 -9999 -9999 -9999 -9999 -9999 -9999 -9999 -9999 -9999 -9999 -9999 -9999 -9999

-9999 -9999 -9999 -9999 -9999 -9999 -9999 -9999 9 9 9 9 9 9 9 9 9 9 7 7 7 7 7 7 7 7 7 7 7 7 7 7 7 7 7 7 7 7 7 -9999 -9999 -9999 -9999 -9999 7 7 7 7 7 7 7 7 -9999 -9999 -9999 -9999 -9999 -9999 -9999 -9999 -9999 -9999 -9999 -9999 -9999 -9999 -9999 -9999 -9999 -9999 -9999 -9999 -9999 -9999 -9999 -9999 -9999 -9999 -9999 -9999 -9999 -9999 -9999

-9999 -9999 -9999 -9999 -9999 -9999 -9999 -9999 9 9 9 9 9 9 7 7 7 7 7 7 7 7 7 7 7 7 7 7 7 7 7 7 7 7 7 7 7 7 7 7 7 7 -9999 -9999 7 7 7 7 7 7 7 7 7 7 7 7 7 7 7 7 7 7 -9999 -9999 -9999 -9999 -9999 -9999 -9999 -9999 -9999 -9999 -9999 -9999 -9999 -9999 -9999 -9999 -9999 -9999 -9999 -9999 -9999

-9999 -9999 -9999 -9999 -9999 -9999 -9999 -9999 9 9 9 9 9 7 7 7 7 7 7 7 7 7 7 7 7 7 7 7 7 7 7 7 7 7 7 7 7 7 7 7 7 7 7 7 7 7 7 7 7 7 7 7 7 7 7 7 7 7 7 7 7 7 -9999 -9999 -9999 -9999 -9999 -9999 -9999 -9999 -9999 -9999 -9999 -9999 -9999 -9999 -9999 -9999 -9999 -9999 -9999 -9999 -9999

-9999 -9999 -9999 -9999 -9999 -9999 -9999 9 9 9 9 9 7 7 7 7 7 7 7 7 7 7 7 7 7 7 7 7 7 7 7 7 7 7 7 7 7 7 7 7 7 7 7 7 7 7 7 7 7 7 7 7 7 7 7 7 7 7 7 -9999 7 7 -9999 -9999 -9999 -9999 -9999 -9999 -9999 -9999 -9999 -9999 -9999 -9999 -9999 -9999 -9999 -9999 -9999 -9999 -9999 -9999 -9999

-9999 -9999 -9999 -9999 -9999 -9999 7 7 9 9 7 7 7 7 7 7 7 7 7 7 7 7 7 7 7 7 7 7 7 7 7 7 7 7 7 7 7 7 7 7 7 7 7 7 7 7 7 7 7 7 7 7 7 7 7 7 7 7 7 7 -9999 7 -9999 -9999 -9999 -9999 -9999 -9999 -9999 -9999 -9999 -9999 -9999 -9999 -9999 -9999 -9999 -9999 -9999 -9999 -9999 -9999 -9999

-9999 -9999 -9999 -9999 7 7 7 7 7 7 7 7 7 7 7 7 7 7 7 7 7 7 7 7 7 7 7 7 7 7 7 7 7 7 7 7 7 7 7 7 7 7 7 7 7 7 7 7 7 7 7 7 7 7 7 7 7 7 7 7 7 -9999 -9999 -9999 -9999 -9999 -9999 -9999 -9999 -9999 -9999 -9999 -9999 -9999 -9999 -9999 -9999 -9999 -9999 -9999 -9999 -9999 -9999

-9999 -9999 -9999 7 7 7 7 7 7 7 7 7 7 7 7 7 7 7 7 7 7 7 7 7 7 7 7 7 7 7 7 7 7 7 7 7 7 7 7 7 7 7 7 7 7 7 7 7 7 7 7 7 7 7 7 7 7 7 7 7 7 -9999 -9999 -9999 -9999 -9999 -9999 -9999 -9999 -9999 -9999 -9999 -9999 -9999 -9999 -9999 -9999 -9999 -9999 -9999 -9999 -9999 -9999

-9999 -9999 7 7 7 7 7 7 7 7 7 7 7 7 7 7 7 7 7 7 7 7 7 7 7 7 7 7 7 7 7 7 7 7 7 7 7 7 7 7 7 7 7 7 7 7 7 7 7 7 7 7 7 7 7 7 7 7 7 7 7 7 -9999 -9999 -9999 -9999 -9999 -9999 -9999 -9999 -9999 -9999 -9999 -9999 -9999 -9999 -9999 -9999 -9999 -9999 -9999 -9999 -9999

-9999 -9999 7 7 7 7 7 7 7 7 7 7 7 7 7 7 7 7 7 7 7 7 7 7 7 7 7 7 7 7 7 7 7 7 7 7 7 7 7 7 7 7 7 7 7 7 7 7 7 7 7 7 7 7 7 7 7 7 7 7 7 7 -9999 -9999 -9999 -9999 -9999 -9999 -9999 -9999 -9999 -9999 -9999 -9999 -9999 -9999 -9999 -9999 -9999 -9999 -9999 -9999 -9999

-9999 7 7 7 7 7 7 7 7 7 7 7 7 7 7 7 7 7 7 7 7 7 7 7 7 7 7 7 7 7 7 7 7 7 7 7 7 7 7 7 7 7 7 7 7 7 7 7 7 7 7 7 7 7 7 7 7 7 7 7 7 7 7 -9999 -9999 -9999 -9999 -9999 -9999 -9999 -9999 -9999 -9999 -9999 -9999 -9999 -9999 -9999 -9999 -9999 -9999 -9999 -9999

-9999 7 7 7 7 7 7 7 7 7 7 7 7 7 7 7 7 7 7 7 7 7 7 7 7 7 7 7 7 7 7 7 7 7 7 7 7 7 7 7 7 7 7 7 7 7 7 7 7 7 7 7 7 7 7 7 7 7 7 7 7 7 7 -9999 -9999 -9999 -9999 -9999 -9999 -9999 -9999 -9999 -9999 -9999 -9999 -9999 -9999 -9999 -9999 -9999 -9999 -9999 -9999

7 7 7 7 7 7 7 7 7 7 7 7 7 7 7 7 7 7 7 7 7 7 7 7 7 7 7 7 7 7 7 7 7 7 7 7 7 7 7 7 7 7 7 7 7 7 7 7 7 7 7 7 7 7 7 7 7 7 7 7 7 7 7 7 -9999 -9999 -9999 -9999 -9999 -9999 -9999 -9999 -9999 -9999 -9999 -9999 -9999 -9999 -9999 -9999 -9999 -9999 -9999

7 7 7 7 7 7 7 7 7 7 7 7 7 7 7 7 7 7 7 7 7 7 7 7 7 7 7 7 7 7 7 7 7 7 7 7 7 7 7 7 7 7 7 7 7 7 7 7 7 7 7 7 7 7 7 7 7 7 7 7 7 7 7 7 7 -9999 -9999 -9999 -9999 -9999 -9999 -9999 -9999 -9999 -9999 -9999 -9999 -9999 -9999 -9999 -9999 -9999 -9999

7 7 7 7 7 7 7 7 7 7 7 7 7 7 7 7 7 7 7 7 7 7 7 7 7 7 7 7 7 7 7 7 7 7 7 7 7 7 7 7 7 7 7 7 7 7 7 7 7 7 7 7 7 7 7 7 7 7 7 7 7 7 7 7 7 -9999 -9999 -9999 -9999 -9999 -9999 -9999 -9999 -9999 -9999 -9999 -9999 -9999 -9999 -9999 -9999 -9999 -9999

-9999 7 7 7 7 7 7 7 7 7 7 7 7 7 7 7 7 7 7 7 7 7 7 7 7 7 7 7 7 7 7 7 7 7 7 7 7 7 7 7 7 7 7 7 7 7 7 7 7 7 7 7 7 7 7 7 7 7 7 7 7 7 7 7 7 -9999 -9999 -9999 -9999 -9999 -9999 -9999 -9999 -9999 -9999 -9999 -9999 -9999 -9999 -9999 -9999 -9999 -9999

-9999 5 7 7 7 7 7 7 5 5 7 7 7 7 7 7 7 5 5 5 5 7 7 7 7 7 7 7 7 7 7 7 7 7 7 7 7 7 7 7 7 7 7 7 7 7 7 7 7 7 7 7 7 7 7 7 7 7 7 7 7 7 7 7 7 -9999 -9999 -9999 -9999 -9999 -9999 -9999 -9999 -9999 -9999 -9999 -9999 -9999 -9999 -9999 -9999 -9999 -9999

-9999 5 5 5 5 5 5 5 5 5 5 5 5 5 5 5 5 5 5 5 5 5 5 5 5 5 7 7 7 7 7 7 7 7 7 7 7 7 7 7 7 7 7 7 7 7 7 7 7 7 7 7 7 7 7 7 7 7 7 7 7 7 7 7 7 7 -9999 -9999 -9999 -9999 -9999 -9999 -9999 -9999 -9999 -9999 -9999 -9999 -9999 -9999 -9999 -9999 -9999

-9999 5 5 5 5 5 5 5 5 5 5 5 5 5 5 5 5 5 5 5 5 5 5 5 5 5 5 5 5 5 5 5 5 5 5 7 7 7 7 7 7 7 7 7 7 7 5 7 7 7 7 7 7 7 7 7 7 7 7 7 7 7 7 7 7 7 7 -9999 -9999 -9999 -9999 -9999 -9999 -9999 -9999 -9999 -9999 -9999 -9999 -9999 -9999 -9999 -9999

5 5 5 5 5 5 5 5 5 5 5 5 5 5 5 5 5 5 5 5 5 5 5 5 5 5 5 5 5 5 5 5 5 5 5 5 5 5 5 5 5 5 5 5 5 5 5 5 5 5 5 5 7 7 7 7 7 7 7 7 7 7 7 7 7 7 5 -9999 -9999 -9999 -9999 -9999 -9999 -9999 -9999 -9999 -9999 -9999 -9999 -9999 -9999 -9999 -9999

6 6 6 5 5 5 5 5 5 5 5 5 5 5 6 6 6 6 6 5 5 5 5 5 5 5 5 5 5 5 5 5 5 5 5 5 5 5 5 5 5 5 5 5 5 5 5 5 5 5 5 5 5 5 5 5 5 5 5 5 5 5 5 5 5 5 5 5 -9999 -9999 -9999 -9999 -9999 -9999 -9999 -9999 -9999 -9999 -9999 -9999 -9999 -9999 -9999

6 6 6 6 6 6 6 6 6 6 6 6 6 6 6 6 6 6 6 6 6 6 6 6 5 5 5 5 5 5 5 5 5 5 5 5 5 5 5 5 5 5 5 5 5 5 5 5 5 5 5 5 5 5 5 5 5 5 5 5 5 5 5 5 5 5 5 5 6 -9999 -9999 -9999 -9999 -9999 -9999 -9999 -9999 -9999 -9999 -9999 -9999 -9999 -9999

6 6 6 6 6 6 6 6 6 6 6 6 6 6 6 6 6 6 6 6 6 6 6 6 6 6 6 6 5 5 5 5 5 5 5 5 5 5 5 5 5 5 5 5 5 5 5 5 5 5 5 5 5 5 5 5 5 5 5 5 5 5 5 5 5 6 6 6 6 6 -9999 -9999 -9999 -9999 -9999 -9999 -9999 -9999 -9999 -9999 -9999 -9999 -9999

6 6 6 6 6 6 6 6 6 6 6 6 6 6 6 6 6 6 6 6 6 6 6 6 6 6 6 6 6 6 6 5 5 5 5 5 5 5 5 5 5 5 5 5 5 6 5 5 5 5 5 5 5 5 5 6 6 5 6 6 6 6 6 6 6 6 3 3 3 6 6 -9999 -9999 -9999 -9999 -9999 -9999 -9999 -9999 -9999 -9999 -9999 -9999

6 6 6 6 6 6 6 6 6 6 6 6 6 6 6 6 6 6 6 6 6 6 6 6 6 6 6 6 6 6 6 6 6 6 6 6 6 6 6 6 6 6 6 6 6 6 6 6 6 6 6 6 6 6 6 6 6 6 6 6 6 6 6 6 3 3 3 3 3 6 6 6 -9999 -9999 -9999 -9999 -9999 -9999 -9999 -9999 -9999 -9999 -9999

-9999 9 9 9 9 9 9 9 9 9 9 9 9 9 9 9 9 9 6 6 6 6 6 6 6 6 6 6 6 6 6 6 6 6 6 6 6 6 6 6 6 6 6 6 6 6 6 6 6 6 6 6 6 6 6 6 6 6 6 6 6 6 6 6 3 3 3 3 3 6 6 6 -9999 -9999 -9999 -9999 -9999 -9999 -9999 -9999 -9999 6 -9999

-9999 -9999 -9999 9 9 9 9 9 9 9 9 9 9 9 9 9 9 9 9 9 9 6 6 6 6 6 9 9 9 9 9 9 9 6 6 6 6 6 6 6 6 6 6 6 6 6 6 6 6 6 6 6 6 6 6 6 6 6 6 6 6 6 6 6 3 3 3 3 6 6 6 6 6 -9999 -9999 6 6 6 6 6 6 6 -9999

-9999 -9999 -9999 9 9 9 9 9 9 9 9 9 9 9 9 9 9 9 9 9 9 9 9 9 9 9 9 9 9 9 9 9 9 9 9 6 6 9 9 9 9 6 6 6 6 6 6 6 6 6 6 6 6 6 6 6 6 6 6 6 6 6 3 3 3 3 12 3 3 6 6 6 6 6 6 6 6 6 6 6 6 -9999 -9999

-9999 -9999 -9999 -9999 9 9 9 9 9 9 9 9 9 9 9 9 9 9 9 9 9 9 9 9 9 9 9 9 9 9 9 9 9 9 9 9 9 9 9 9 9 9 9 6 6 6 6 6 6 6 6 6 6 6 6 6 6 6 6 6 6 6 3 12 12 12 12 3 3 12 3 3 6 6 6 6 6 6 6 6 6 -9999 -9999

-9999 -9999 -9999 -9999 9 9 9 9 9 9 9 9 9 9 1 1 9 9 9 9 9 9 9 9 9 9 9 9 9 9 9 9 9 9 9 9 9 9 9 9 9 9 9 9 9 9 9 9 9 9 9 9 9 6 6 6 6 6 6 6 6 6 3 12 12 12 3 3 12 12 6 6 6 6 6 6 6 6 6 6 -9999 -9999 -9999

-9999 -9999 -9999 -9999 -9999 1 1 1 1 1 9 9 9 1 1 1 1 9 9 9 9 9 9 9 9 9 9 9 9 9 9 9 9 9 9 9 9 9 9 9 9 9 9 9 9 9 9 9 9 9 9 9 9 9 6 6 6 6 6 6 6 6 3 12 12 12 3 12 12 6 6 6 6 6 6 6 6 6 6 6 -9999 -9999 -9999

-9999 -9999 -9999 -9999 -9999 -9999 -9999 1 1 1 1 9 1 1 1 1 1 9 9 9 9 9 9 9 9 9 1 1 1 9 9 9 9 9 9 9 9 9 9 9 9 9 9 9 9 9 9 9 9 9 9 9 9 9 9 6 6 6 6 6 6 6 3 3 3 3 12 3 6 6 6 6 6 6 6 6 6 6 6 -9999 -9999 -9999 -9999

-9999 -9999 -9999 -9999 -9999 -9999 -9999 -9999 1 1 1 1 1 1 1 1 1 1 9 9 -9999 -9999 -9999 -9999 -9999 -9999 1 1 1 1 1 1 1 1 9 9 9 9 9 9 9 9 9 9 9 9 9 9 9 9 9 9 9 9 9 9 6 6 6 6 6 6 9 9 6 3 3 6 6 6 6 6 6 6 6 6 6 6 6 -9999 -9999 -9999 -9999

-9999 -9999 -9999 -9999 -9999 -9999 -9999 -9999 -9999 -9999 1 1 1 -9999 -9999 -9999 -9999 9 -9999 -9999 -9999 -9999 -9999 -9999 -9999 -9999 -9999 1 1 1 1 1 1 9 9 9 9 1 1 1 1 1 1 1 1 1 1 1 9 1 1 1 9 9 9 9 6 6 6 6 6 6 9 9 6 6 6 6 6 6 6 6 6 6 6 6 6 6 -9999 -9999 -9999 -9999 -9999

-9999 -9999 -9999 -9999 -9999 -9999 -9999 -9999 -9999 -9999 -9999 -9999 -9999 -9999 -9999 -9999 -9999 -9999 -9999 -9999 -9999 -9999 -9999 -9999 -9999 -9999 -9999 -9999 -9999 -9999 -9999 1 1 1 1 1 1 1 1 1 1 1 1 1 1 1 1 1 1 1 1 9 9 9 9 9 9 6 6 6 6 6 9 9 6 6 6 6 6 6 6 6 6 6 6 6 6 -9999 -9999 -9999 -9999 -9999 -9999

-9999 -9999 -9999 -9999 -9999 -9999 -9999 -9999 -9999 -9999 -9999 -9999 -9999 -9999 -9999 -9999 -9999 -9999 -9999 -9999 -9999 -9999 -9999 -9999 -9999 -9999 -9999 -9999 -9999 -9999 -9999 -9999 1 1 1 1 1 1 1 1 1 1 1 1 1 1 1 1 1 1 1 1 1 9 9 9 9 6 6 6 6 9 9 9 6 6 6 6 6 6 6 6 6 6 6 6 6 -9999 -9999 -9999 -9999 -9999 -9999

-9999 -9999 -9999 -9999 -9999 -9999 -9999 -9999 -9999 -9999 -9999 -9999 -9999 -9999 -9999 -9999 -9999 -9999 -9999 -9999 -9999 -9999 -9999 -9999 -9999 -9999 -9999 -9999 -9999 -9999 -9999 -9999 1 1 1 1 1 1 1 1 1 1 1 1 1 1 1 1 1 1 1 1 1 1 9 9 9 6 6 6 6 9 9 9 4 4 6 6 6 6 6 6 6 6 6 6 -9999 -9999 -9999 -9999 -9999 -9999 -9999

-9999 -9999 -9999 -9999 -9999 -9999 -9999 -9999 -9999 -9999 -9999 -9999 -9999 -9999 -9999 -9999 -9999 -9999 -9999 -9999 -9999 -9999 -9999 -9999 -9999 -9999 -9999 -9999 -9999 -9999 -9999 -9999 1 1 1 1 1 1 1 1 1 1 1 1 1 1 1 1 1 1 1 1 1 1 1 9 9 9 6 6 6 9 9 9 4 4 4 6 6 6 6 6 6 6 -9999 -9999 -9999 -9999 -9999 -9999 -9999 -9999 -9999

-9999 -9999 -9999 -9999 -9999 -9999 -9999 -9999 -9999 -9999 -9999 -9999 -9999 -9999 -9999 -9999 -9999 -9999 -9999 -9999 -9999 -9999 -9999 -9999 -9999 -9999 -9999 -9999 -9999 -9999 -9999 -9999 1 1 1 1 1 1 1 1 1 1 1 1 1 1 1 1 1 1 1 1 1 1 9 9 9 9 6 6 6 9 9 12 12 4 4 4 4 6 6 6 6 -9999 -9999 -9999 -9999 -9999 -9999 -9999 -9999 -9999 -9999

-9999 -9999 -9999 -9999 -9999 -9999 -9999 -9999 -9999 -9999 -9999 -9999 -9999 -9999 -9999 -9999 -9999 -9999 -9999 -9999 -9999 -9999 -9999 -9999 -9999 -9999 -9999 -9999 -9999 -9999 -9999 1 1 1 1 1 1 1 1 1 1 1 1 1 1 1 1 1 1 1 1 1 1 1 9 9 9 9 9 6 -9999 12 12 12 12 4 4 4 4 4 4 4 -9999 -9999 -9999 -9999 -9999 -9999 -9999 -9999 -9999 -9999 -9999

-9999 -9999 -9999 -9999 -9999 -9999 -9999 -9999 -9999 -9999 -9999 -9999 -9999 -9999 -9999 -9999 -9999 -9999 -9999 -9999 -9999 -9999 -9999 -9999 -9999 -9999 -9999 -9999 -9999 -9999 -9999 1 1 1 9 9 1 1 1 1 1 1 1 1 1 1 1 1 1 1 1 1 1 1 9 9 9 9 -9999 -9999 -9999 9 12 12 12 4 4 4 4 4 4 -9999 -9999 -9999 -9999 -9999 -9999 -9999 -9999 -9999 -9999 -9999 -9999

-9999 -9999 -9999 -9999 -9999 -9999 -9999 -9999 -9999 -9999 -9999 -9999 -9999 -9999 -9999 -9999 -9999 -9999 -9999 -9999 -9999 -9999 -9999 -9999 -9999 -9999 -9999 -9999 -9999 -9999 -9999 1 1 1 9 9 9 9 1 1 1 1 1 1 1 1 1 1 1 1 1 1 1 12 12 12 12 9 -9999 -9999 -9999 9 9 12 12 4 4 4 4 4 -9999 -9999 -9999 -9999 -9999 -9999 -9999 -9999 -9999 -9999 -9999 -9999 -9999

-9999 -9999 -9999 -9999 -9999 -9999 -9999 -9999 -9999 -9999 -9999 -9999 -9999 -9999 -9999 -9999 -9999 -9999 -9999 -9999 -9999 -9999 -9999 -9999 -9999 -9999 -9999 -9999 -9999 -9999 -9999 -9999 1 1 1 9 9 9 1 1 1 1 1 1 1 1 1 1 1 1 1 1 1 1 12 12 9 9 -9999 9 9 9 9 12 12 12 4 4 4 -9999 -9999 -9999 -9999 -9999 -9999 -9999 -9999 -9999 -9999 -9999 -9999 -9999 -9999

-9999 -9999 -9999 -9999 -9999 -9999 -9999 -9999 -9999 -9999 -9999 -9999 -9999 -9999 -9999 -9999 -9999 -9999 -9999 -9999 -9999 -9999 -9999 -9999 -9999 -9999 -9999 -9999 -9999 -9999 -9999 -9999 -9999 1 9 9 9 1 1 1 1 1 1 1 1 1 1 1 1 1 1 1 1 1 12 12 9 9 9 3 3 9 9 12 12 12 4 4 -9999 -9999 -9999 -9999 -9999 -9999 -9999 -9999 -9999 -9999 -9999 -9999 -9999 -9999 -9999

-9999 -9999 -9999 -9999 -9999 -9999 -9999 -9999 -9999 -9999 -9999 -9999 -9999 -9999 -9999 -9999 -9999 -9999 -9999 -9999 -9999 -9999 -9999 -9999 -9999 -9999 -9999 -9999 -9999 -9999 -9999 -9999 -9999 -9999 9 9 1 1 1 1 1 1 1 1 1 1 1 1 1 1 1 1 1 1 1 -9999 9 9 9 3 3 9 9 9 9 4 4 4 -9999 -9999 -9999 -9999 -9999 -9999 -9999 -9999 -9999 -9999 -9999 -9999 -9999 -9999 -9999

-9999 -9999 -9999 -9999 -9999 -9999 -9999 -9999 -9999 -9999 -9999 -9999 -9999 -9999 -9999 -9999 -9999 -9999 -9999 -9999 -9999 -9999 -9999 -9999 -9999 -9999 -9999 -9999 -9999 -9999 -9999 -9999 -9999 -9999 -9999 9 9 1 1 1 1 1 1 1 1 1 1 1 1 1 1 1 1 1 1 -9999 9 9 9 3 3 9 9 9 4 4 4 -9999 -9999 -9999 -9999 -9999 -9999 -9999 -9999 -9999 -9999 -9999 -9999 -9999 -9999 -9999 -9999

-9999 -9999 -9999 -9999 -9999 -9999 -9999 -9999 -9999 -9999 -9999 -9999 -9999 -9999 -9999 -9999 -9999 -9999 -9999 -9999 -9999 -9999 -9999 -9999 -9999 -9999 -9999 -9999 -9999 -9999 -9999 -9999 -9999 -9999 -9999 9 9 1 1 1 1 1 1 1 1 1 1 1 1 9 9 9 1 1 1 -9999 9 9 3 3 3 9 9 9 4 4 4 4 -9999 -9999 -9999 -9999 -9999 -9999 -9999 -9999 -9999 -9999 -9999 -9999 -9999 -9999 -9999

-9999 -9999 -9999 -9999 -9999 -9999 -9999 -9999 -9999 -9999 -9999 -9999 -9999 -9999 -9999 -9999 -9999 -9999 -9999 -9999 -9999 -9999 -9999 -9999 -9999 -9999 -9999 -9999 -9999 -9999 -9999 -9999 -9999 -9999 -9999 9 9 9 9 1 1 1 1 1 1 9 9 9 9 9 9 9 9 3 3 3 -9999 9 3 3 3 3 9 9 4 4 4 -9999 -9999 -9999 -9999 -9999 -9999 -9999 -9999 -9999 -9999 -9999 -9999 -9999 -9999 -9999 -9999

-9999 -9999 -9999 -9999 -9999 -9999 -9999 -9999 -9999 -9999 -9999 -9999 -9999 -9999 -9999 -9999 -9999 -9999 -9999 -9999 -9999 -9999 -9999 -9999 -9999 -9999 -9999 -9999 -9999 -9999 -9999 -9999 -9999 -9999 -9999 -9999 9 9 9 9 9 9 9 9 9 9 9 9 9 9 9 9 9 3 3 3 9 9 3 3 3 3 9 4 4 4 4 -9999 -9999 -9999 -9999 -9999 -9999 -9999 -9999 -9999 -9999 -9999 -9999 -9999 -9999 -9999 -9999

-9999 -9999 -9999 -9999 -9999 -9999 -9999 -9999 -9999 -9999 -9999 -9999 -9999 -9999 -9999 -9999 -9999 -9999 -9999 -9999 -9999 -9999 -9999 -9999 -9999 -9999 -9999 -9999 -9999 -9999 -9999 -9999 -9999 -9999 -9999 -9999 9 9 9 9 9 9 9 9 9 9 9 9 9 9 9 9 9 3 3 3 3 3 3 3 3 4 4 4 4 4 4 -9999 -9999 -9999 -9999 -9999 -9999 -9999 -9999 -9999 -9999 -9999 -9999 -9999 -9999 -9999 -9999

-9999 -9999 -9999 -9999 -9999 -9999 -9999 -9999 -9999 -9999 -9999 -9999 -9999 -9999 -9999 -9999 -9999 -9999 -9999 -9999 -9999 -9999 -9999 -9999 -9999 -9999 -9999 -9999 -9999 -9999 -9999 -9999 -9999 -9999 -9999 -9999 9 9 9 9 9 9 9 9 9 9 9 9 9 9 9 9 3 3 3 3 3 3 6 6 3 4 4 4 4 4 4 4 -9999 -9999 -9999 -9999 -9999 -9999 -9999 -9999 -9999 -9999 -9999 -9999 -9999 -9999 -9999

-9999 -9999 -9999 -9999 -9999 -9999 -9999 -9999 -9999 -9999 -9999 -9999 -9999 -9999 -9999 -9999 -9999 -9999 -9999 -9999 -9999 -9999 -9999 -9999 -9999 -9999 -9999 -9999 -9999 -9999 -9999 -9999 -9999 -9999 -9999 -9999 9 9 9 9 9 9 9 9 9 9 9 9 9 9 3 3 3 3 3 3 3 6 6 6 6 -9999 4 4 4 4 4 4 -9999 -9999 -9999 -9999 -9999 -9999 -9999 -9999 -9999 -9999 -9999 -9999 -9999 -9999 -9999

-9999 -9999 -9999 -9999 -9999 -9999 -9999 -9999 -9999 -9999 -9999 -9999 -9999 -9999 -9999 -9999 -9999 -9999 -9999 -9999 -9999 -9999 -9999 -9999 -9999 -9999 -9999 -9999 -9999 -9999 -9999 -9999 -9999 -9999 -9999 -9999 -9999 9 9 9 9 9 9 9 9 3 3 3 3 3 3 3 3 3 3 3 3 6 6 6 6 -9999 4 4 4 4 4 4 4 -9999 -9999 -9999 -9999 -9999 -9999 -9999 -9999 -9999 -9999 -9999 -9999 -9999 -9999

-9999 -9999 -9999 -9999 -9999 -9999 -9999 -9999 -9999 -9999 -9999 -9999 -9999 -9999 -9999 -9999 -9999 -9999 -9999 -9999 -9999 -9999 -9999 -9999 -9999 -9999 -9999 -9999 -9999 -9999 -9999 -9999 -9999 -9999 -9999 -9999 -9999 9 9 9 9 9 9 9 3 3 3 3 3 3 3 3 3 3 3 3 6 6 6 6 6 -9999 4 4 4 4 4 4 4 -9999 -9999 -9999 -9999 -9999 -9999 -9999 -9999 -9999 -9999 -9999 -9999 -9999 -9999

-9999 -9999 -9999 -9999 -9999 -9999 -9999 -9999 -9999 -9999 -9999 -9999 -9999 -9999 -9999 -9999 -9999 -9999 -9999 -9999 -9999 -9999 -9999 -9999 -9999 -9999 -9999 -9999 -9999 -9999 -9999 -9999 -9999 -9999 -9999 -9999 9 9 9 9 9 9 9 9 3 3 3 3 3 3 3 3 3 3 6 6 6 6 6 6 4 -9999 4 4 4 4 4 4 4 -9999 -9999 -9999 -9999 -9999 -9999 -9999 -9999 -9999 -9999 -9999 -9999 -9999 -9999

-9999 -9999 -9999 -9999 -9999 -9999 -9999 -9999 -9999 -9999 -9999 -9999 -9999 -9999 -9999 -9999 -9999 -9999 -9999 -9999 -9999 -9999 -9999 -9999 -9999 -9999 -9999 -9999 -9999 -9999 -9999 -9999 -9999 -9999 -9999 9 9 9 9 9 9 9 9 3 3 3 3 3 3 3 3 3 3 3 6 6 6 6 6 6 4 -9999 4 4 4 4 3 3 3 -9999 -9999 -9999 -9999 -9999 -9999 -9999 -9999 -9999 -9999 -9999 -9999 -9999 -9999

-9999 -9999 -9999 -9999 -9999 -9999 -9999 -9999 -9999 -9999 -9999 -9999 -9999 -9999 -9999 -9999 -9999 -9999 -9999 -9999 -9999 -9999 -9999 -9999 -9999 -9999 -9999 -9999 -9999 -9999 -9999 -9999 -9999 -9999 -9999 9 9 9 9 9 9 3 3 3 3 3 3 3 3 3 3 3 3 3 6 6 6 6 6 6 4 4 -9999 3 3 3 3 3 3 -9999 -9999 -9999 -9999 -9999 -9999 -9999 -9999 -9999 -9999 -9999 -9999 -9999 -9999

-9999 -9999 -9999 -9999 -9999 -9999 -9999 -9999 -9999 -9999 -9999 -9999 -9999 -9999 -9999 -9999 -9999 -9999 -9999 -9999 -9999 -9999 -9999 -9999 -9999 -9999 -9999 -9999 -9999 -9999 -9999 -9999 -9999 -9999 -9999 9 9 9 9 9 3 3 3 3 3 3 3 3 3 3 3 3 3 3 6 6 6 6 6 4 4 4 4 3 3 3 3 3 3 -9999 -9999 -9999 -9999 -9999 -9999 -9999 -9999 -9999 -9999 -9999 -9999 -9999 -9999

-9999 -9999 -9999 -9999 -9999 -9999 -9999 -9999 -9999 -9999 -9999 -9999 -9999 -9999 -9999 -9999 -9999 -9999 -9999 -9999 -9999 -9999 -9999 -9999 -9999 -9999 -9999 -9999 -9999 -9999 -9999 -9999 -9999 -9999 5 5 5 9 9 9 3 3 3 3 3 3 3 3 3 3 3 3 3 6 6 6 6 6 6 6 4 4 3 3 3 3 3 3 -9999 -9999 -9999 -9999 -9999 -9999 -9999 -9999 -9999 -9999 -9999 -9999 -9999 -9999 -9999

-9999 -9999 -9999 -9999 -9999 -9999 -9999 -9999 -9999 -9999 -9999 -9999 -9999 -9999 -9999 -9999 -9999 -9999 -9999 -9999 -9999 -9999 -9999 -9999 -9999 -9999 -9999 -9999 -9999 -9999 -9999 -9999 -9999 -9999 5 5 9 9 9 9 3 3 3 3 3 3 3 3 3 3 3 3 3 6 6 6 6 6 6 6 4 4 4 3 3 3 3 3 -9999 -9999 -9999 -9999 -9999 -9999 -9999 -9999 -9999 -9999 -9999 -9999 -9999 -9999 -9999

-9999 -9999 -9999 -9999 -9999 -9999 -9999 -9999 -9999 -9999 -9999 -9999 -9999 -9999 -9999 -9999 -9999 -9999 -9999 -9999 -9999 -9999 -9999 -9999 -9999 -9999 -9999 -9999 -9999 -9999 -9999 -9999 -9999 -9999 5 5 9 9 9 6 6 6 6 6 6 6 6 6 3 3 3 3 3 6 6 6 6 6 6 6 6 4 4 4 3 -9999 -9999 -9999 -9999 -9999 -9999 -9999 -9999 -9999 -9999 -9999 -9999 -9999 -9999 -9999 -9999 -9999 -9999

-9999 -9999 -9999 -9999 -9999 -9999 -9999 -9999 -9999 -9999 -9999 -9999 -9999 -9999 -9999 -9999 -9999 -9999 -9999 -9999 -9999 -9999 -9999 -9999 -9999 -9999 -9999 -9999 -9999 -9999 -9999 -9999 -9999 -9999 -9999 5 5 5 6 6 6 6 6 6 6 6 6 6 6 6 3 6 6 6 6 6 6 6 6 6 6 4 4 4 -9999 -9999 -9999 -9999 -9999 -9999 -9999 -9999 -9999 -9999 -9999 -9999 -9999 -9999 -9999 -9999 -9999 -9999 -9999

-9999 -9999 -9999 -9999 -9999 -9999 -9999 -9999 -9999 -9999 -9999 -9999 -9999 -9999 -9999 -9999 -9999 -9999 -9999 -9999 -9999 -9999 -9999 -9999 -9999 -9999 -9999 -9999 -9999 -9999 -9999 -9999 -9999 -9999 -9999 7 5 5 5 5 6 6 6 6 6 6 6 6 6 6 6 6 6 6 6 6 6 6 6 6 6 4 4 -9999 -9999 -9999 -9999 -9999 -9999 -9999 -9999 -9999 -9999 -9999 -9999 -9999 -9999 -9999 -9999 -9999 -9999 -9999 -9999

-9999 -9999 -9999 -9999 -9999 -9999 -9999 -9999 -9999 -9999 -9999 -9999 -9999 -9999 -9999 -9999 -9999 -9999 -9999 -9999 -9999 -9999 -9999 -9999 -9999 -9999 -9999 -9999 -9999 -9999 -9999 -9999 -9999 -9999 -9999 -9999 7 5 5 5 5 6 6 6 5 6 6 6 6 6 6 6 6 6 6 6 6 6 6 6 6 4 -9999 -9999 -9999 -9999 -9999 -9999 -9999 -9999 -9999 -9999 -9999 -9999 -9999 -9999 -9999 -9999 -9999 -9999 -9999 -9999 -9999

-9999 -9999 -9999 -9999 -9999 -9999 -9999 -9999 -9999 -9999 -9999 -9999 -9999 -9999 -9999 -9999 -9999 -9999 -9999 -9999 -9999 -9999 -9999 -9999 -9999 -9999 -9999 -9999 -9999 -9999 -9999 -9999 -9999 -9999 -9999 -9999 7 5 5 5 5 5 5 5 5 5 5 5 6 6 6 6 6 6 6 6 6 6 6 6 6 4 -9999 -9999 -9999 -9999 -9999 -9999 -9999 -9999 -9999 -9999 -9999 -9999 -9999 -9999 -9999 -9999 -9999 -9999 -9999 -9999 -9999

-9999 -9999 -9999 -9999 -9999 -9999 -9999 -9999 -9999 -9999 -9999 -9999 -9999 -9999 -9999 -9999 -9999 -9999 -9999 -9999 -9999 -9999 -9999 -9999 -9999 -9999 -9999 -9999 -9999 -9999 -9999 -9999 -9999 -9999 -9999 -9999 -9999 7 5 5 5 5 5 5 5 5 5 5 5 6 6 6 6 6 6 6 6 6 6 6 6 4 4 -9999 -9999 -9999 -9999 -9999 -9999 -9999 -9999 -9999 -9999 -9999 -9999 -9999 -9999 -9999 -9999 -9999 -9999 -9999 -9999

-9999 -9999 -9999 -9999 -9999 -9999 -9999 -9999 -9999 -9999 -9999 -9999 -9999 -9999 -9999 -9999 -9999 -9999 -9999 -9999 -9999 -9999 -9999 -9999 -9999 -9999 -9999 -9999 -9999 -9999 -9999 -9999 -9999 -9999 -9999 -9999 -9999 -9999 5 5 5 5 5 5 5 5 5 5 5 6 6 6 6 6 6 6 6 6 6 6 6 6 4 -9999 -9999 -9999 -9999 -9999 -9999 -9999 -9999 -9999 -9999 -9999 -9999 -9999 -9999 -9999 -9999 -9999 -9999 -9999 -9999

-9999 -9999 -9999 -9999 -9999 -9999 -9999 -9999 -9999 -9999 -9999 -9999 -9999 -9999 -9999 -9999 -9999 -9999 -9999 -9999 -9999 -9999 -9999 -9999 -9999 -9999 -9999 -9999 -9999 -9999 -9999 -9999 -9999 -9999 -9999 -9999 -9999 -9999 5 5 5 5 5 5 5 5 5 5 5 6 6 6 6 6 6 6 6 6 6 6 6 4 4 -9999 -9999 -9999 -9999 -9999 -9999 -9999 -9999 -9999 -9999 -9999 -9999 -9999 -9999 -9999 -9999 -9999 -9999 -9999 -9999

-9999 -9999 -9999 -9999 -9999 -9999 -9999 -9999 -9999 -9999 -9999 -9999 -9999 -9999 -9999 -9999 -9999 -9999 -9999 -9999 -9999 -9999 -9999 -9999 -9999 -9999 -9999 -9999 -9999 -9999 -9999 -9999 -9999 -9999 -9999 -9999 -9999 -9999 7 5 5 5 5 5 5 5 5 5 5 6 6 6 6 6 6 6 6 6 6 6 12 4 4 -9999 -9999 -9999 -9999 -9999 -9999 -9999 -9999 -9999 -9999 -9999 -9999 -9999 -9999 -9999 -9999 -9999 -9999 -9999 -9999

-9999 -9999 -9999 -9999 -9999 -9999 -9999 -9999 -9999 -9999 -9999 -9999 -9999 -9999 -9999 -9999 -9999 -9999 -9999 -9999 -9999 -9999 -9999 -9999 -9999 -9999 -9999 -9999 -9999 -9999 -9999 -9999 -9999 -9999 -9999 -9999 -9999 -9999 7 5 5 5 5 5 5 5 5 5 5 6 6 6 6 6 6 6 6 6 6 12 12 -9999 -9999 -9999 -9999 -9999 -9999 -9999 -9999 -9999 -9999 -9999 -9999 -9999 -9999 -9999 -9999 -9999 -9999 -9999 -9999 -9999 -9999

-9999 -9999 -9999 -9999 -9999 -9999 -9999 -9999 -9999 -9999 -9999 -9999 -9999 -9999 -9999 -9999 -9999 -9999 -9999 -9999 -9999 -9999 -9999 -9999 -9999 -9999 -9999 -9999 -9999 -9999 -9999 -9999 -9999 -9999 -9999 -9999 -9999 -9999 7 5 5 5 5 5 5 5 5 5 5 6 6 6 6 6 6 6 6 6 12 -9999 -9999 -9999 -9999 -9999 -9999 -9999 -9999 -9999 -9999 -9999 -9999 -9999 -9999 -9999 -9999 -9999 -9999 -9999 -9999 -9999 -9999 -9999 -9999

-9999 -9999 -9999 -9999 -9999 -9999 -9999 -9999 -9999 -9999 -9999 -9999 -9999 -9999 -9999 -9999 -9999 -9999 -9999 -9999 -9999 -9999 -9999 -9999 -9999 -9999 -9999 -9999 -9999 -9999 -9999 -9999 -9999 -9999 -9999 -9999 -9999 -9999 7 7 5 5 5 5 5 5 5 5 5 6 6 6 6 6 6 6 6 6 12 12 -9999 -9999 -9999 -9999 -9999 -9999 -9999 -9999 -9999 -9999 -9999 -9999 -9999 -9999 -9999 -9999 -9999 -9999 -9999 -9999 -9999 -9999 -9999

-9999 -9999 -9999 -9999 -9999 -9999 -9999 -9999 -9999 -9999 -9999 -9999 -9999 -9999 -9999 -9999 -9999 -9999 -9999 -9999 -9999 -9999 -9999 -9999 -9999 -9999 -9999 -9999 -9999 -9999 -9999 -9999 -9999 -9999 -9999 -9999 -9999 -9999 -9999 7 7 5 5 5 5 5 5 5 5 6 6 6 6 6 6 6 6 6 12 -9999 -9999 -9999 -9999 -9999 -9999 -9999 -9999 -9999 -9999 -9999 -9999 -9999 -9999 -9999 -9999 -9999 -9999 -9999 -9999 -9999 -9999 -9999 -9999

-9999 -9999 -9999 -9999 -9999 -9999 -9999 -9999 -9999 -9999 -9999 -9999 -9999 -9999 -9999 -9999 -9999 -9999 -9999 -9999 -9999 -9999 -9999 -9999 -9999 -9999 -9999 -9999 -9999 -9999 -9999 -9999 -9999 -9999 -9999 -9999 -9999 -9999 -9999 7 7 5 5 5 5 5 5 5 5 6 6 6 6 6 6 6 6 6 12 -9999 -9999 -9999 -9999 -9999 -9999 -9999 -9999 -9999 -9999 -9999 -9999 -9999 -9999 -9999 -9999 -9999 -9999 -9999 -9999 -9999 -9999 -9999 -9999

-9999 -9999 -9999 -9999 -9999 -9999 -9999 -9999 -9999 -9999 -9999 -9999 -9999 -9999 -9999 -9999 -9999 -9999 -9999 -9999 -9999 -9999 -9999 -9999 -9999 -9999 -9999 -9999 -9999 -9999 -9999 -9999 -9999 -9999 -9999 -9999 -9999 -9999 -9999 -9999 7 7 5 5 5 5 5 5 6 6 6 6 6 6 6 6 6 12 -9999 -9999 -9999 -9999 -9999 -9999 -9999 -9999 -9999 -9999 -9999 -9999 -9999 -9999 -9999 -9999 -9999 -9999 -9999 -9999 -9999 -9999 -9999 -9999 -9999

-9999 -9999 -9999 -9999 -9999 -9999 -9999 -9999 -9999 -9999 -9999 -9999 -9999 -9999 -9999 -9999 -9999 -9999 -9999 -9999 -9999 -9999 -9999 -9999 -9999 -9999 -9999 -9999 -9999 -9999 -9999 -9999 -9999 -9999 -9999 -9999 -9999 -9999 -9999 -9999 -9999 7 5 5 5 5 5 5 6 6 6 6 6 6 6 6 6 -9999 -9999 -9999 -9999 -9999 -9999 -9999 -9999 -9999 -9999 -9999 -9999 -9999 -9999 -9999 -9999 -9999 -9999 -9999 -9999 -9999 -9999 -9999 -9999 -9999 -9999

-9999 -9999 -9999 -9999 -9999 -9999 -9999 -9999 -9999 -9999 -9999 -9999 -9999 -9999 -9999 -9999 -9999 -9999 -9999 -9999 -9999 -9999 -9999 -9999 -9999 -9999 -9999 -9999 -9999 -9999 -9999 -9999 -9999 -9999 -9999 -9999 -9999 -9999 -9999 -9999 -9999 7 7 5 5 5 5 5 6 6 6 6 6 6 6 6 12 -9999 -9999 -9999 -9999 -9999 -9999 -9999 -9999 -9999 -9999 -9999 -9999 -9999 -9999 -9999 -9999 -9999 -9999 -9999 -9999 -9999 -9999 -9999 -9999 -9999 -9999

-9999 -9999 -9999 -9999 -9999 -9999 -9999 -9999 -9999 -9999 -9999 -9999 -9999 -9999 -9999 -9999 -9999 -9999 -9999 -9999 -9999 -9999 -9999 -9999 -9999 -9999 -9999 -9999 -9999 -9999 -9999 -9999 -9999 -9999 -9999 -9999 -9999 -9999 -9999 -9999 -9999 -9999 7 5 5 5 5 6 6 6 6 6 6 6 6 12 -9999 -9999 -9999 -9999 -9999 -9999 -9999 -9999 -9999 -9999 -9999 -9999 -9999 -9999 -9999 -9999 -9999 -9999 -9999 -9999 -9999 -9999 -9999 -9999 -9999 -9999 -9999

-9999 -9999 -9999 -9999 -9999 -9999 -9999 -9999 -9999 -9999 -9999 -9999 -9999 -9999 -9999 -9999 -9999 -9999 -9999 -9999 -9999 -9999 -9999 -9999 -9999 -9999 -9999 -9999 -9999 -9999 -9999 -9999 -9999 -9999 -9999 -9999 -9999 -9999 -9999 -9999 -9999 -9999 7 5 5 3 3 6 6 6 6 6 6 6 12 -9999 -9999 -9999 -9999 -9999 -9999 -9999 -9999 -9999 -9999 -9999 -9999 -9999 -9999 -9999 -9999 -9999 -9999 -9999 -9999 -9999 -9999 -9999 -9999 -9999 -9999 -9999 -9999

-9999 -9999 -9999 -9999 -9999 -9999 -9999 -9999 -9999 -9999 -9999 -9999 -9999 -9999 -9999 -9999 -9999 -9999 -9999 -9999 -9999 -9999 -9999 -9999 -9999 -9999 -9999 -9999 -9999 -9999 -9999 -9999 -9999 -9999 -9999 -9999 -9999 -9999 -9999 -9999 -9999 -9999 9 3 3 3 3 6 6 6 6 6 6 -9999 -9999 -9999 -9999 -9999 -9999 -9999 -9999 -9999 -9999 -9999 -9999 -9999 -9999 -9999 -9999 -9999 -9999 -9999 -9999 -9999 -9999 -9999 -9999 -9999 -9999 -9999 -9999 -9999 -9999

-9999 -9999 -9999 -9999 -9999 -9999 -9999 -9999 -9999 -9999 -9999 -9999 -9999 -9999 -9999 -9999 -9999 -9999 -9999 -9999 -9999 -9999 -9999 -9999 -9999 -9999 -9999 -9999 -9999 -9999 -9999 -9999 -9999 -9999 -9999 -9999 -9999 -9999 -9999 -9999 -9999 -9999 9 9 9 6 6 -9999 -9999 6 -9999 -9999 -9999 -9999 -9999 -9999 -9999 -9999 -9999 -9999 -9999 -9999 -9999 -9999 -9999 -9999 -9999 -9999 -9999 -9999 -9999 -9999 -9999 -9999 -9999 -9999 -9999 -9999 -9999 -9999 -9999 -9999 -9999

## roughness.asc

ncols 83

nrows 87

xllcorner -16.733229949463

yllcorner -35.237187

cellsize 0.83

NODATA_value -9999

-9999 -9999 -9999 -9999 -9999 -9999 -9999 -9999 -9999 -9999 -9999 -9999 -9999 -9999 -9999 -9999 -9999 -9999 -9999 -9999 -9999 -9999 -9999 -9999 -9999 -9999 -9999 -9999 -9999 -9999 -9999 0.5 -9999 -9999 -9999 -9999 -9999 -9999 -9999 -9999 -9999 -9999 -9999 -9999 -9999 -9999 -9999 -9999 -9999 -9999 -9999 -9999 -9999 -9999 -9999 -9999 -9999 -9999 -9999 -9999 -9999 -9999 -9999 -9999 -9999 -9999 -9999 -9999 -9999 -9999 -9999 -9999 -9999 -9999 -9999 -9999 -9999 -9999 -9999 -9999 -9999 -9999 -9999

-9999 -9999 -9999 -9999 -9999 -9999 -9999 -9999 -9999 -9999 -9999 -9999 -9999 -9999 -9999 -9999 -9999 -9999 -9999 -9999 -9999 0.5 0.5 0.5 0.5 0.5 0.5 0.5 0.5 0.5 0.5 0.5 0.1 -9999 -9999 -9999 -9999 -9999 -9999 -9999 -9999 -9999 -9999 -9999 -9999 -9999 -9999 -9999 -9999 -9999 -9999 -9999 -9999 -9999 -9999 -9999 -9999 -9999 -9999 -9999 -9999 -9999 -9999 -9999 -9999 -9999 -9999 -9999 -9999 -9999 -9999 -9999 -9999 -9999 -9999 -9999 -9999 -9999 -9999 -9999 -9999 -9999 -9999

-9999 -9999 -9999 -9999 -9999 -9999 -9999 -9999 -9999 -9999 -9999 -9999 -9999 0.5 -9999 -9999 -9999 -9999 -9999 0.5 0.5 0.1 0.5 0.5 0.5 0.5 0.5 0.5 0.5 0.5 0.5 0.5 0.1 -9999 -9999 -9999 -9999 -9999 -9999 -9999 -9999 -9999 -9999 -9999 -9999 -9999 -9999 -9999 -9999 -9999 -9999 -9999 -9999 -9999 -9999 -9999 -9999 -9999 -9999 -9999 -9999 -9999 -9999 -9999 -9999 -9999 -9999 -9999 -9999 -9999 -9999 -9999 -9999 -9999 -9999 -9999 -9999 -9999 -9999 -9999 -9999 -9999 -9999

-9999 -9999 -9999 -9999 -9999 -9999 -9999 -9999 -9999 -9999 -9999 -9999 0.5 0.5 0.5 0.5 0.5 0.5 0.1 0.1 0.1 0.5 0.5 0.5 0.5 0.5 0.1 0.1 0.1 0.5 0.5 0.1 0.1 -9999 -9999 -9999 -9999 -9999 -9999 -9999 -9999 -9999 -9999 -9999 -9999 -9999 -9999 -9999 -9999 -9999 -9999 -9999 -9999 -9999 -9999 -9999 -9999 -9999 -9999 -9999 -9999 -9999 -9999 -9999 -9999 -9999 -9999 -9999 -9999 -9999 -9999 -9999 -9999 -9999 -9999 -9999 -9999 -9999 -9999 -9999 -9999 -9999 -9999

-9999 -9999 -9999 -9999 -9999 -9999 -9999 -9999 -9999 -9999 -9999 -9999 0.5 0.5 0.5 0.5 0.5 0.5 0.5 0.5 0.5 0.5 0.1 0.1 0.1 0.1 0.1 0.1 0.1 0.1 0.1 0.1 -9999 -9999 -9999 -9999 -9999 -9999 -9999 -9999 -9999 -9999 -9999 -9999 -9999 -9999 -9999 -9999 -9999 -9999 -9999 -9999 -9999 -9999 -9999 -9999 -9999 -9999 -9999 -9999 -9999 -9999 -9999 -9999 -9999 -9999 -9999 -9999 -9999 -9999 -9999 -9999 -9999 -9999 -9999 -9999 -9999 -9999 -9999 -9999 -9999 -9999 -9999

-9999 -9999 -9999 -9999 -9999 -9999 -9999 -9999 -9999 -9999 0.5 0.5 0.9 0.5 0.5 0.5 0.5 0.5 0.5 0.5 0.1 0.1 0.1 0.1 0.1 0.1 0.1 0.1 0.1 0.1 0.1 0.1 0.5 0.1 -9999 -9999 -9999 -9999 -9999 -9999 -9999 -9999 -9999 -9999 -9999 -9999 -9999 -9999 -9999 -9999 -9999 -9999 -9999 -9999 -9999 -9999 -9999 -9999 -9999 -9999 -9999 -9999 -9999 -9999 -9999 -9999 -9999 -9999 -9999 -9999 -9999 -9999 -9999 -9999 -9999 -9999 -9999 -9999 -9999 -9999 -9999 -9999 -9999

-9999 -9999 -9999 -9999 -9999 -9999 -9999 -9999 -9999 0.5 0.9 0.9 0.5 0.5 0.5 0.1 0.1 0.5 0.1 0.1 0.1 0.1 0.1 0.1 0.1 0.1 0.1 0.1 0.1 0.1 0.1 0.1 0.1 0.1 0.1 0.5 0.5 0.1 -9999 -9999 -9999 -9999 -9999 -9999 0.5 0.1 0.1 0.1 -9999 -9999 -9999 -9999 -9999 -9999 -9999 -9999 -9999 -9999 -9999 -9999 -9999 -9999 -9999 -9999 -9999 -9999 -9999 -9999 -9999 -9999 -9999 -9999 -9999 -9999 -9999 -9999 -9999 -9999 -9999 -9999 -9999 -9999 -9999

-9999 -9999 -9999 -9999 -9999 -9999 -9999 -9999 0.5 0.9 0.9 0.5 0.5 0.5 0.1 0.1 0.1 0.1 0.1 0.1 0.1 0.1 0.1 0.1 0.1 0.1 0.1 0.1 0.1 0.1 0.1 0.1 0.1 0.1 0.1 0.1 0.1 0.1 0.1 -9999 -9999 -9999 -9999 -9999 0.1 0.1 0.1 0.1 0.1 0.1 0.1 0.1 -9999 -9999 -9999 -9999 -9999 -9999 -9999 -9999 -9999 -9999 -9999 -9999 -9999 -9999 -9999 -9999 -9999 -9999 -9999 -9999 -9999 -9999 -9999 -9999 -9999 -9999 -9999 -9999 -9999 -9999 -9999

-9999 -9999 -9999 -9999 -9999 -9999 -9999 -9999 0.5 0.5 0.5 0.5 0.5 0.1 0.1 0.1 0.1 0.1 0.1 0.1 0.1 0.1 0.1 0.1 0.1 0.1 0.1 0.1 0.1 0.1 0.1 0.1 0.1 0.1 0.1 0.1 0.1 0.1 0.1 0.1 0.1 0.1 -9999 -9999 0.1 0.1 0.1 0.1 0.1 0.1 0.1 0.1 0.1 0.1 0.1 0.1 0.1 0.5 0.5 0.5 0.5 0.5 -9999 -9999 -9999 -9999 -9999 -9999 -9999 -9999 -9999 -9999 -9999 -9999 -9999 -9999 -9999 -9999 -9999 -9999 -9999 -9999 -9999

-9999 -9999 -9999 -9999 -9999 -9999 -9999 -9999 0.5 0.5 0.1 0.1 0.1 0.1 0.1 0.1 0.1 0.1 0.1 0.1 0.1 0.1 0.1 0.1 0.1 0.1 0.1 0.1 0.1 0.1 0.1 0.1 0.1 0.1 0.1 0.1 0.1 0.1 0.1 0.1 0.1 0.1 0.1 0.1 0.1 0.1 0.1 0.1 0.1 0.1 0.1 0.1 0.1 0.1 0.1 0.1 0.1 0.5 0.5 0.5 0.5 0.9 -9999 -9999 -9999 -9999 -9999 -9999 -9999 -9999 -9999 -9999 -9999 -9999 -9999 -9999 -9999 -9999 -9999 -9999 -9999 -9999 -9999

-9999 -9999 -9999 -9999 -9999 -9999 -9999 0.5 0.1 0.1 0.1 0.1 0.1 0.1 0.1 0.1 0.1 0.1 0.1 0.1 0.1 0.1 0.1 0.1 0.1 0.1 0.1 0.1 0.1 0.1 0.1 0.1 0.1 0.1 0.1 0.1 0.1 0.1 0.1 0.1 0.1 0.1 0.1 0.1 0.1 0.1 0.1 0.1 0.1 0.1 0.1 0.1 0.1 0.1 0.1 0.1 0.1 0.1 0.5 -9999 0.9 0.5 -9999 -9999 -9999 -9999 -9999 -9999 -9999 -9999 -9999 -9999 -9999 -9999 -9999 -9999 -9999 -9999 -9999 -9999 -9999 -9999 -9999

-9999 -9999 -9999 -9999 -9999 -9999 0.1 0.1 0.1 0.1 0.1 0.1 0.1 0.1 0.1 0.1 0.1 0.1 0.1 0.1 0.1 0.1 0.1 0.1 0.1 0.1 0.1 0.1 0.1 0.1 0.1 0.1 0.1 0.1 0.1 0.1 0.1 0.1 0.1 0.1 0.1 0.5 0.1 0.1 0.1 0.1 0.1 0.1 0.1 0.1 0.1 0.1 0.1 0.1 0.1 0.1 0.1 0.1 0.1 0.5 -9999 -9999 -9999 -9999 -9999 -9999 -9999 -9999 -9999 -9999 -9999 -9999 -9999 -9999 -9999 -9999 -9999 -9999 -9999 -9999 -9999 -9999 -9999

-9999 -9999 -9999 -9999 0.1 0.1 0.1 0.1 0.1 0.1 0.1 0.1 0.1 0.1 0.1 0.1 0.1 0.1 0.1 0.1 0.1 0.1 0.1 0.1 0.1 0.5 0.5 0.5 0.5 0.5 0.5 0.1 0.1 0.1 0.1 0.1 0.1 0.1 0.1 0.1 0.1 0.1 0.1 0.1 0.1 0.1 0.1 0.1 0.1 0.1 0.1 0.1 0.1 0.1 0.1 0.1 0.1 0.1 0.5 0.5 0.5 -9999 -9999 -9999 -9999 -9999 -9999 -9999 -9999 -9999 -9999 -9999 -9999 -9999 -9999 -9999 -9999 -9999 -9999 -9999 -9999 -9999 -9999

-9999 -9999 -9999 0.1 0.1 0.1 0.1 0.1 0.1 0.1 0.1 0.1 0.1 0.1 0.1 0.1 0.1 0.1 0.1 0.1 0.1 0.1 0.1 0.1 0.5 0.5 0.5 0.5 0.5 0.5 0.5 0.5 0.1 0.5 0.1 0.1 0.1 0.1 0.1 0.1 0.1 0.1 0.1 0.1 0.1 0.1 0.1 0.1 0.1 0.1 0.1 0.1 0.1 0.1 0.1 0.1 0.5 0.1 0.5 0.5 0.1 -9999 -9999 -9999 -9999 -9999 -9999 -9999 -9999 -9999 -9999 -9999 -9999 -9999 -9999 -9999 -9999 -9999 -9999 -9999 -9999 -9999 -9999

-9999 -9999 0.1 0.1 0.1 0.1 0.1 0.1 0.1 0.1 0.1 0.1 0.1 0.1 0.1 0.1 0.1 0.1 0.1 0.1 0.1 0.1 0.1 0.1 0.5 0.5 0.5 0.5 0.5 0.5 0.5 0.5 0.5 0.5 0.1 0.1 0.1 0.1 0.1 0.1 0.5 0.1 0.1 0.1 0.1 0.1 0.1 0.1 0.1 0.1 0.5 0.5 0.5 0.1 0.1 0.1 0.1 0.1 0.5 0.1 0.1 0.5 -9999 -9999 -9999 -9999 -9999 -9999 -9999 -9999 -9999 -9999 -9999 -9999 -9999 -9999 -9999 -9999 -9999 -9999 -9999 -9999 -9999

-9999 -9999 0.1 0.1 0.1 0.1 0.1 0.1 0.1 0.1 0.1 0.1 0.1 0.1 0.1 0.1 0.1 0.1 0.1 0.1 0.1 0.1 0.1 0.1 0.5 0.1 0.5 0.5 0.5 0.5 0.1 0.5 0.5 0.5 0.1 0.1 0.1 0.1 0.1 0.1 0.1 0.1 0.1 0.1 0.1 0.1 0.1 0.1 0.1 0.1 0.5 0.5 0.5 0.1 0.1 0.1 0.1 0.1 0.1 0.1 0.1 0.5 -9999 -9999 -9999 -9999 -9999 -9999 -9999 -9999 -9999 -9999 -9999 -9999 -9999 -9999 -9999 -9999 -9999 -9999 -9999 -9999 -9999

-9999 0.1 0.1 0.1 0.1 0.1 0.1 0.1 0.1 0.1 0.1 0.1 0.1 0.1 0.1 0.1 0.1 0.1 0.1 0.1 0.1 0.1 0.1 0.1 0.1 0.5 0.5 0.5 0.5 0.5 0.5 0.1 0.1 0.5 0.1 0.1 0.1 0.1 0.5 0.1 0.1 0.1 0.5 0.5 0.1 0.1 0.1 0.1 0.1 0.1 0.5 0.5 0.1 0.1 0.1 0.1 0.1 0.1 0.1 0.1 0.1 0.1 0.5 -9999 -9999 -9999 -9999 -9999 -9999 -9999 -9999 -9999 -9999 -9999 -9999 -9999 -9999 -9999 -9999 -9999 -9999 -9999 -9999

-9999 0.1 0.1 0.1 0.1 0.1 0.1 0.1 0.1 0.1 0.1 0.1 0.1 0.1 0.1 0.1 0.1 0.1 0.1 0.1 0.1 0.1 0.1 0.1 0.1 0.5 0.5 0.5 0.1 0.1 0.5 0.1 0.1 0.1 0.1 0.1 0.1 0.1 0.1 0.1 0.1 0.5 0.5 0.5 0.1 0.1 0.1 0.1 0.1 0.1 0.1 0.1 0.1 0.1 0.1 0.1 0.1 0.1 0.1 0.1 0.1 0.1 0.5 -9999 -9999 -9999 -9999 -9999 -9999 -9999 -9999 -9999 -9999 -9999 -9999 -9999 -9999 -9999 -9999 -9999 -9999 -9999 -9999

0.1 0.1 0.1 0.1 0.1 0.1 0.1 0.1 0.1 0.1 0.1 0.1 0.1 0.1 0.1 0.1 0.1 0.1 0.1 0.1 0.1 0.1 0.1 0.1 0.1 0.1 0.1 0.1 0.1 0.1 0.1 0.5 0.1 0.1 0.1 0.1 0.1 0.1 0.1 0.5 0.5 0.5 0.5 0.5 0.1 0.1 0.1 0.1 0.1 0.5 0.1 0.1 0.1 0.1 0.1 0.1 0.1 0.1 0.1 0.1 0.1 0.1 0.5 0.5 -9999 -9999 -9999 -9999 -9999 -9999 -9999 -9999 -9999 -9999 -9999 -9999 -9999 -9999 -9999 -9999 -9999 -9999 -9999

0.1 0.1 0.1 0.1 0.1 0.1 0.1 0.1 0.1 0.1 0.1 0.1 0.1 0.1 0.1 0.1 0.1 0.1 0.1 0.1 0.1 0.1 0.1 0.1 0.1 0.1 0.1 0.1 0.1 0.1 0.5 0.1 0.1 0.1 0.1 0.1 0.1 0.1 0.1 0.5 0.5 0.5 0.9 0.5 0.1 0.1 0.1 0.1 0.1 0.1 0.1 0.1 0.1 0.1 0.1 0.1 0.1 0.1 0.1 0.1 0.1 0.1 0.1 0.1 0.5 -9999 -9999 -9999 -9999 -9999 -9999 -9999 -9999 -9999 -9999 -9999 -9999 -9999 -9999 -9999 -9999 -9999 -9999

-9999 0.1 0.1 0.1 0.1 0.1 0.1 0.1 0.1 0.1 0.1 0.1 0.1 0.1 0.1 0.1 0.1 0.1 0.1 0.1 0.1 0.1 0.1 0.1 0.1 0.1 0.1 0.1 0.1 0.5 0.5 0.5 0.1 0.1 0.1 0.1 0.1 0.1 0.1 0.1 0.5 0.5 0.9 0.5 0.1 0.1 0.1 0.1 0.1 0.1 0.1 0.1 0.1 0.1 0.1 0.1 0.1 0.1 0.1 0.1 0.1 0.1 0.1 0.1 0.5 -9999 -9999 -9999 -9999 -9999 -9999 -9999 -9999 -9999 -9999 -9999 -9999 -9999 -9999 -9999 -9999 -9999 -9999

-9999 0.1 0.1 0.1 0.1 0.1 0.1 0.1 0.1 0.1 0.1 0.1 0.1 0.1 0.1 0.1 0.1 0.1 0.1 0.1 0.1 0.1 0.1 0.1 0.1 0.1 0.1 0.1 0.1 0.5 0.5 0.5 0.1 0.1 0.1 0.1 0.1 0.1 0.1 0.1 0.1 0.1 0.5 0.1 0.1 0.1 0.1 0.1 0.1 0.1 0.1 0.1 0.1 0.1 0.1 0.1 0.1 0.1 0.1 0.1 0.1 0.1 0.1 0.1 0.5 -9999 -9999 -9999 -9999 -9999 -9999 -9999 -9999 -9999 -9999 -9999 -9999 -9999 -9999 -9999 -9999 -9999 -9999

-9999 0.1 0.1 0.1 0.1 0.1 0.5 0.1 0.1 0.1 0.1 0.1 0.1 0.1 0.1 0.1 0.1 0.1 0.1 0.1 0.1 0.1 0.1 0.1 0.1 0.1 0.1 0.1 0.1 0.5 0.5 0.5 0.1 0.1 0.1 0.1 0.1 0.1 0.1 0.1 0.1 0.1 0.1 0.1 0.1 0.1 0.1 0.5 0.1 0.1 0.1 0.1 0.1 0.1 0.1 0.1 0.1 0.1 0.1 0.1 0.1 0.1 0.1 0.1 0.5 -9999 -9999 -9999 -9999 -9999 -9999 -9999 -9999 -9999 -9999 -9999 -9999 -9999 -9999 -9999 -9999 -9999 -9999

-9999 0.1 0.1 0.1 0.1 0.1 0.1 0.1 0.1 0.1 0.1 0.1 0.1 0.1 0.1 0.1 0.1 0.1 0.1 0.1 0.1 0.1 0.1 0.1 0.1 0.1 0.1 0.1 0.1 0.1 0.5 0.5 0.1 0.1 0.1 0.1 0.1 0.1 0.1 0.1 0.1 0.1 0.1 0.1 0.1 0.1 0.5 0.5 0.1 0.1 0.1 0.1 0.1 0.1 0.1 0.1 0.1 0.1 0.1 0.1 0.1 0.1 0.1 0.1 0.5 0.5 -9999 -9999 -9999 -9999 -9999 -9999 -9999 -9999 -9999 -9999 -9999 -9999 -9999 -9999 -9999 -9999 -9999

-9999 0.1 0.1 0.1 0.1 0.1 0.1 0.1 0.1 0.1 0.1 0.1 0.1 0.1 0.1 0.1 0.1 0.1 0.1 0.1 0.1 0.1 0.1 0.1 0.1 0.1 0.1 0.1 0.1 0.1 0.1 0.1 0.1 0.1 0.1 0.1 0.1 0.1 0.1 0.1 0.1 0.1 0.1 0.1 0.1 0.1 0.5 0.5 0.1 0.1 0.1 0.1 0.1 0.1 0.1 0.1 0.1 0.5 0.1 0.1 0.1 0.1 0.1 0.1 0.1 0.5 0.9 -9999 -9999 -9999 -9999 -9999 -9999 -9999 -9999 -9999 -9999 -9999 -9999 -9999 -9999 -9999 -9999

0.1 0.1 0.1 0.1 0.1 0.1 0.1 0.1 0.1 0.1 0.1 0.1 0.1 0.1 0.1 0.1 0.1 0.1 0.1 0.1 0.1 0.1 0.1 0.1 0.1 0.1 0.1 0.1 0.1 0.1 0.1 0.1 0.1 0.1 0.1 0.1 0.1 0.1 0.1 0.1 0.1 0.1 0.1 0.1 0.1 0.1 0.5 0.1 0.1 0.1 0.5 0.5 0.1 0.1 0.1 0.1 0.1 0.1 0.1 0.1 0.1 0.1 0.1 0.1 0.1 0.5 0.9 -9999 -9999 -9999 -9999 -9999 -9999 -9999 -9999 -9999 -9999 -9999 -9999 -9999 -9999 -9999 -9999

0.1 0.1 0.1 0.1 0.1 0.1 0.1 0.1 0.1 0.1 0.1 0.1 0.1 0.1 0.1 0.5 0.1 0.1 0.1 0.1 0.1 0.1 0.1 0.1 0.1 0.1 0.1 0.1 0.1 0.1 0.1 0.1 0.1 0.1 0.1 0.1 0.1 0.1 0.1 0.1 0.1 0.1 0.1 0.1 0.1 0.5 0.1 0.1 0.1 0.1 0.5 0.5 0.1 0.1 0.1 0.1 0.1 0.1 0.1 0.1 0.1 0.1 0.1 0.1 0.5 0.5 0.5 0.9 -9999 -9999 -9999 -9999 -9999 -9999 -9999 -9999 -9999 -9999 -9999 -9999 -9999 -9999 -9999

0.1 0.1 0.1 0.1 0.1 0.1 0.1 0.1 0.1 0.1 0.1 0.1 0.1 0.1 0.1 0.1 0.1 0.1 0.1 0.1 0.1 0.1 0.1 0.1 0.1 0.1 0.1 0.1 0.1 0.1 0.1 0.1 0.1 0.1 0.1 0.1 0.1 0.1 0.1 0.1 0.1 0.1 0.1 0.1 0.1 0.1 0.1 0.1 0.5 0.5 0.5 0.1 0.1 0.1 0.1 0.1 0.1 0.1 0.1 0.1 0.1 0.1 0.1 0.1 0.5 0.5 0.5 0.9 0.5 -9999 -9999 -9999 -9999 -9999 -9999 -9999 -9999 -9999 -9999 -9999 -9999 -9999 -9999

0.1 0.1 0.1 0.1 0.1 0.1 0.1 0.1 0.1 0.1 0.1 0.1 0.1 0.1 0.1 0.1 0.1 0.1 0.1 0.1 0.1 0.1 0.1 0.1 0.1 0.1 0.1 0.1 0.1 0.1 0.1 0.1 0.1 0.1 0.1 0.1 0.1 0.1 0.1 0.1 0.1 0.1 0.1 0.1 0.1 0.1 0.1 0.1 0.5 0.5 0.5 0.1 0.1 0.1 0.1 0.1 0.1 0.1 0.1 0.1 0.1 0.1 0.1 0.5 0.5 0.9 0.5 0.9 0.5 0.5 -9999 -9999 -9999 -9999 -9999 -9999 -9999 -9999 -9999 -9999 -9999 -9999 -9999

0.1 0.1 0.1 0.1 0.5 0.5 0.5 0.1 0.1 0.1 0.1 0.1 0.1 0.1 0.1 0.1 0.1 0.1 0.1 0.1 0.1 0.1 0.1 0.1 0.1 0.1 0.1 0.1 0.1 0.1 0.1 0.1 0.1 0.1 0.1 0.1 0.1 0.1 0.1 0.1 0.1 0.1 0.1 0.1 0.1 0.1 0.1 0.5 0.5 0.5 0.1 0.1 0.1 0.1 0.1 0.1 0.1 0.5 0.1 0.1 0.1 0.1 0.1 0.5 0.5 0.5 0.5 0.9 0.5 0.5 0.5 -9999 -9999 -9999 -9999 -9999 -9999 -9999 -9999 -9999 -9999 -9999 -9999

0.1 0.1 0.1 0.1 0.5 0.5 0.5 0.1 0.1 0.1 0.1 0.1 0.1 0.1 0.1 0.1 0.1 0.1 0.1 0.1 0.1 0.1 0.1 0.1 0.1 0.1 0.1 0.1 0.1 0.1 0.1 0.1 0.1 0.1 0.1 0.1 0.1 0.1 0.1 0.1 0.1 0.1 0.1 0.1 0.1 0.1 0.1 0.1 0.1 0.1 0.1 0.1 0.1 0.1 0.1 0.1 0.1 0.5 0.1 0.1 0.1 0.1 0.5 0.5 0.5 0.5 0.5 0.9 0.5 0.5 0.5 0.5 -9999 -9999 -9999 -9999 -9999 -9999 -9999 -9999 -9999 -9999 -9999

-9999 -9999 0.1 0.5 0.5 0.5 0.5 0.1 0.1 0.1 0.1 0.1 0.1 0.1 0.1 0.1 0.1 0.1 0.1 0.1 0.1 0.5 0.1 0.1 0.1 0.1 0.1 0.1 0.1 0.1 0.5 0.1 0.1 0.1 0.1 0.5 0.5 0.1 0.1 0.1 0.1 0.1 0.1 0.1 0.1 0.1 0.1 0.1 0.1 0.1 0.1 0.1 0.1 0.1 0.1 0.1 0.1 0.1 0.1 0.1 0.1 0.5 0.5 0.5 0.5 0.5 0.9 0.9 0.5 0.1 0.5 0.5 -9999 -9999 -9999 -9999 -9999 -9999 -9999 -9999 -9999 0.5 -9999

-9999 -9999 -9999 0.1 0.5 0.5 0.1 0.1 0.1 0.1 0.1 0.1 0.1 0.1 0.1 0.1 0.1 0.1 0.1 0.1 0.1 0.1 0.1 0.1 0.1 0.1 0.1 0.1 0.5 0.1 0.5 0.5 0.1 0.5 0.5 0.5 0.5 0.1 0.1 0.1 0.1 0.1 0.1 0.1 0.1 0.1 0.1 0.1 0.1 0.1 0.1 0.1 0.1 0.1 0.1 0.1 0.1 0.1 0.1 0.1 0.5 0.5 0.5 0.5 0.5 0.5 0.5 0.9 0.5 0.5 0.5 0.5 0.5 -9999 -9999 0.5 0.5 0.5 0.1 0.1 0.5 0.5 -9999

-9999 -9999 -9999 -9999 0.1 0.5 0.5 0.1 0.5 0.1 0.1 0.1 0.1 0.1 0.1 0.1 0.1 0.1 0.1 0.1 0.5 0.1 0.1 0.1 0.1 0.1 0.1 0.1 0.5 0.5 0.5 0.5 0.1 0.5 0.5 0.5 0.1 0.1 0.1 0.1 0.1 0.1 0.1 0.1 0.1 0.1 0.1 0.5 0.1 0.1 0.1 0.1 0.1 0.1 0.1 0.1 0.1 0.1 0.1 0.1 0.5 0.5 0.5 0.5 0.5 0.5 0.5 0.9 0.5 0.5 0.5 0.5 0.5 0.1 0.1 0.1 0.1 0.5 0.5 0.1 0.5 -9999 -9999

-9999 -9999 -9999 -9999 0.5 0.1 0.1 0.1 0.5 0.5 0.1 0.1 0.1 0.1 0.1 0.1 0.1 0.1 0.1 0.1 0.5 0.1 0.1 0.1 0.1 0.1 0.1 0.5 0.1 0.1 0.1 0.1 0.1 0.5 0.5 0.5 0.5 0.5 0.5 0.1 0.1 0.1 0.1 0.1 0.1 0.1 0.1 0.1 0.1 0.1 0.1 0.1 0.1 0.1 0.1 0.1 0.1 0.1 0.1 0.1 0.1 0.5 0.5 0.5 0.5 0.5 0.5 0.9 0.5 0.5 0.5 0.5 0.5 0.1 0.1 0.1 0.1 0.5 0.5 0.1 0.9 -9999 -9999

-9999 -9999 -9999 -9999 -9999 0.5 0.5 0.1 0.1 0.5 0.5 0.1 0.1 0.1 0.1 0.1 0.1 0.1 0.1 0.1 0.5 0.1 0.1 0.1 0.1 0.1 0.1 0.5 0.1 0.1 0.1 0.1 0.5 0.5 0.5 0.5 0.5 0.5 0.5 0.5 0.1 0.1 0.1 0.1 0.1 0.1 0.1 0.1 0.1 0.1 0.1 0.1 0.1 0.1 0.1 0.1 0.1 0.1 0.1 0.1 0.1 0.5 0.5 0.5 0.5 0.5 0.5 0.9 0.5 0.5 0.5 0.5 0.1 0.1 0.1 0.1 0.1 0.1 0.1 0.5 -9999 -9999 -9999

-9999 -9999 -9999 -9999 -9999 -9999 0.9 0.5 0.1 0.1 0.1 0.1 0.1 0.1 0.1 0.1 0.1 0.1 0.1 0.1 0.5 0.5 0.5 0.5 0.5 0.5 0.1 0.1 0.1 0.1 0.5 0.5 0.5 0.5 0.5 0.5 0.5 0.5 0.5 0.5 0.1 0.1 0.1 0.1 0.1 0.1 0.1 0.1 0.1 0.1 0.1 0.1 0.1 0.1 0.1 0.1 0.1 0.1 0.1 0.1 0.1 0.5 0.5 0.5 0.5 0.5 0.5 0.9 0.9 0.5 0.5 0.5 0.5 0.5 0.1 0.1 0.1 0.1 0.1 0.9 -9999 -9999 -9999

-9999 -9999 -9999 -9999 -9999 -9999 -9999 0.9 0.5 0.1 0.1 0.1 0.5 0.5 0.5 0.5 0.5 0.1 0.1 0.5 0.9 0.9 0.9 0.9 0.9 0.5 0.1 0.1 0.1 0.1 0.5 0.5 0.5 0.5 0.1 0.1 0.1 0.1 0.1 0.1 0.1 0.1 0.1 0.1 0.1 0.1 0.1 0.1 0.1 0.1 0.1 0.1 0.1 0.1 0.1 0.1 0.1 0.1 0.1 0.1 0.1 0.5 0.5 0.5 0.9 0.5 0.5 0.5 0.5 0.5 0.5 0.5 0.5 0.1 0.1 0.1 0.1 0.1 0.9 -9999 -9999 -9999 -9999

-9999 -9999 -9999 -9999 -9999 -9999 -9999 -9999 -9999 0.5 0.5 0.5 0.9 0.9 0.9 0.9 0.5 0.5 0.9 -9999 -9999 -9999 -9999 -9999 -9999 -9999 -9999 0.1 0.1 0.1 0.5 0.5 0.5 0.5 0.1 0.1 0.1 0.1 0.1 0.1 0.1 0.1 0.1 0.1 0.1 0.1 0.1 0.1 0.1 0.1 0.1 0.1 0.1 0.1 0.1 0.1 0.1 0.5 0.5 0.5 0.5 0.5 0.5 0.5 0.5 0.5 0.5 0.5 0.5 0.5 0.5 0.1 0.1 0.1 0.1 0.1 0.1 0.5 0.9 -9999 -9999 -9999 -9999

-9999 -9999 -9999 -9999 -9999 -9999 -9999 -9999 -9999 -9999 -9999 -9999 -9999 -9999 -9999 -9999 -9999 0.9 -9999 -9999 -9999 -9999 -9999 -9999 -9999 -9999 -9999 -9999 0.5 0.5 0.9 0.5 0.5 0.5 0.1 0.1 0.1 0.1 0.1 0.1 0.1 0.1 0.1 0.1 0.1 0.1 0.1 0.1 0.1 0.1 0.1 0.1 0.1 0.1 0.1 0.1 0.1 0.5 0.5 0.5 0.5 0.5 0.5 0.5 0.5 0.5 0.5 0.5 0.5 0.5 0.1 0.1 0.1 0.1 0.1 0.1 0.5 0.9 -9999 -9999 -9999 -9999 -9999

-9999 -9999 -9999 -9999 -9999 -9999 -9999 -9999 -9999 -9999 -9999 -9999 -9999 -9999 -9999 -9999 -9999 -9999 -9999 -9999 -9999 -9999 -9999 -9999 -9999 -9999 -9999 -9999 -9999 -9999 -9999 -9999 0.5 0.5 0.1 0.1 0.1 0.1 0.1 0.1 0.1 0.1 0.1 0.1 0.1 0.1 0.1 0.1 0.1 0.1 0.1 0.1 0.1 0.1 0.1 0.1 0.5 0.5 0.5 0.1 0.5 0.5 0.5 0.5 0.5 0.5 0.5 0.5 0.1 0.1 0.5 0.1 0.5 0.1 0.1 0.5 0.9 -9999 -9999 -9999 -9999 -9999 -9999

-9999 -9999 -9999 -9999 -9999 -9999 -9999 -9999 -9999 -9999 -9999 -9999 -9999 -9999 -9999 -9999 -9999 -9999 -9999 -9999 -9999 -9999 -9999 -9999 -9999 -9999 -9999 -9999 -9999 -9999 -9999 -9999 0.5 0.1 0.1 0.1 0.1 0.1 0.1 0.1 0.1 0.1 0.1 0.1 0.1 0.1 0.1 0.1 0.1 0.1 0.1 0.1 0.1 0.1 0.1 0.1 0.5 0.5 0.1 0.1 0.1 0.5 0.5 0.5 0.5 0.5 0.1 0.1 0.1 0.1 0.1 0.1 0.1 0.1 0.5 0.9 0.9 -9999 -9999 -9999 -9999 -9999 -9999

-9999 -9999 -9999 -9999 -9999 -9999 -9999 -9999 -9999 -9999 -9999 -9999 -9999 -9999 -9999 -9999 -9999 -9999 -9999 -9999 -9999 -9999 -9999 -9999 -9999 -9999 -9999 -9999 -9999 -9999 -9999 -9999 0.5 0.1 0.1 0.1 0.1 0.1 0.1 0.1 0.1 0.1 0.1 0.1 0.1 0.1 0.1 0.1 0.1 0.1 0.1 0.1 0.1 0.1 0.1 0.5 0.5 0.5 0.1 0.1 0.1 0.5 0.9 0.5 0.5 0.5 0.1 0.1 0.1 0.1 0.1 0.1 0.1 0.5 0.9 -9999 -9999 -9999 -9999 -9999 -9999 -9999 -9999

-9999 -9999 -9999 -9999 -9999 -9999 -9999 -9999 -9999 -9999 -9999 -9999 -9999 -9999 -9999 -9999 -9999 -9999 -9999 -9999 -9999 -9999 -9999 -9999 -9999 -9999 -9999 -9999 -9999 -9999 -9999 -9999 0.5 0.1 0.1 0.1 0.1 0.1 0.1 0.1 0.1 0.1 0.1 0.1 0.1 0.1 0.1 0.1 0.1 0.1 0.1 0.1 0.1 0.1 0.1 0.9 0.5 0.5 0.1 0.1 0.1 0.5 0.9 0.5 0.5 0.5 0.5 0.1 0.1 0.1 0.1 0.1 0.9 0.9 -9999 -9999 -9999 -9999 -9999 -9999 -9999 -9999 -9999

-9999 -9999 -9999 -9999 -9999 -9999 -9999 -9999 -9999 -9999 -9999 -9999 -9999 -9999 -9999 -9999 -9999 -9999 -9999 -9999 -9999 -9999 -9999 -9999 -9999 -9999 -9999 -9999 -9999 -9999 -9999 -9999 0.1 0.5 0.1 0.1 0.1 0.1 0.1 0.1 0.1 0.1 0.1 0.1 0.1 0.1 0.1 0.1 0.1 0.1 0.1 0.1 0.1 0.5 0.5 0.9 0.5 0.1 0.1 0.1 0.1 0.5 0.5 0.5 0.9 0.5 0.5 0.1 0.1 0.1 0.1 0.9 0.9 -9999 -9999 -9999 -9999 -9999 -9999 -9999 -9999 -9999 -9999

-9999 -9999 -9999 -9999 -9999 -9999 -9999 -9999 -9999 -9999 -9999 -9999 -9999 -9999 -9999 -9999 -9999 -9999 -9999 -9999 -9999 -9999 -9999 -9999 -9999 -9999 -9999 -9999 -9999 -9999 -9999 -9999 0.1 0.5 0.5 0.5 0.1 0.1 0.1 0.1 0.1 0.1 0.1 0.1 0.1 0.1 0.1 0.1 0.1 0.1 0.1 0.1 0.5 0.5 0.5 0.5 0.5 0.1 0.1 0.1 -9999 0.5 0.5 0.5 0.5 0.5 0.5 0.1 0.1 0.1 0.9 0.9 -9999 -9999 -9999 -9999 -9999 -9999 -9999 -9999 -9999 -9999 -9999

-9999 -9999 -9999 -9999 -9999 -9999 -9999 -9999 -9999 -9999 -9999 -9999 -9999 -9999 -9999 -9999 -9999 -9999 -9999 -9999 -9999 -9999 -9999 -9999 -9999 -9999 -9999 -9999 -9999 -9999 -9999 0.1 0.1 0.5 0.5 0.5 0.1 0.1 0.1 0.1 0.1 0.1 0.1 0.1 0.1 0.1 0.1 0.1 0.1 0.1 0.1 0.5 0.5 0.5 0.5 0.5 0.5 0.5 -9999 -9999 -9999 0.5 0.5 0.5 0.5 0.5 0.5 0.1 0.1 0.5 0.9 -9999 -9999 -9999 -9999 -9999 -9999 -9999 -9999 -9999 -9999 -9999 -9999

-9999 -9999 -9999 -9999 -9999 -9999 -9999 -9999 -9999 -9999 -9999 -9999 -9999 -9999 -9999 -9999 -9999 -9999 -9999 -9999 -9999 -9999 -9999 -9999 -9999 -9999 -9999 -9999 -9999 -9999 -9999 0.5 0.1 0.5 0.5 0.1 0.1 0.1 0.1 0.1 0.1 0.1 0.1 0.1 0.1 0.1 0.1 0.1 0.1 0.1 0.1 0.5 0.5 0.5 0.5 0.5 0.5 0.5 -9999 -9999 -9999 0.1 0.5 0.5 0.5 0.5 0.5 0.1 0.1 0.9 -9999 -9999 -9999 -9999 -9999 -9999 -9999 -9999 -9999 -9999 -9999 -9999 -9999

-9999 -9999 -9999 -9999 -9999 -9999 -9999 -9999 -9999 -9999 -9999 -9999 -9999 -9999 -9999 -9999 -9999 -9999 -9999 -9999 -9999 -9999 -9999 -9999 -9999 -9999 -9999 -9999 -9999 -9999 -9999 -9999 0.5 0.5 0.5 0.5 0.1 0.1 0.1 0.5 0.1 0.1 0.1 0.1 0.1 0.1 0.1 0.1 0.1 0.1 0.1 0.1 0.5 0.5 0.9 0.5 0.5 0.1 -9999 0.1 0.1 0.5 0.5 0.5 0.9 0.5 0.5 0.5 0.5 -9999 -9999 -9999 -9999 -9999 -9999 -9999 -9999 -9999 -9999 -9999 -9999 -9999 -9999

-9999 -9999 -9999 -9999 -9999 -9999 -9999 -9999 -9999 -9999 -9999 -9999 -9999 -9999 -9999 -9999 -9999 -9999 -9999 -9999 -9999 -9999 -9999 -9999 -9999 -9999 -9999 -9999 -9999 -9999 -9999 -9999 -9999 -9999 0.1 0.5 0.5 0.1 0.5 0.1 0.1 0.1 0.1 0.1 0.1 0.1 0.1 0.1 0.1 0.1 0.1 0.1 0.1 0.5 0.5 0.5 0.5 0.1 0.1 0.1 0.1 0.5 0.5 0.5 0.5 0.5 0.5 0.5 -9999 -9999 -9999 -9999 -9999 -9999 -9999 -9999 -9999 -9999 -9999 -9999 -9999 -9999 -9999

-9999 -9999 -9999 -9999 -9999 -9999 -9999 -9999 -9999 -9999 -9999 -9999 -9999 -9999 -9999 -9999 -9999 -9999 -9999 -9999 -9999 -9999 -9999 -9999 -9999 -9999 -9999 -9999 -9999 -9999 -9999 -9999 -9999 -9999 0.5 0.1 0.5 0.5 0.1 0.1 0.1 0.1 0.1 0.1 0.1 0.1 0.1 0.1 0.1 0.1 0.1 0.1 0.1 0.1 0.5 -9999 0.5 0.1 0.1 0.1 0.1 0.5 0.5 0.5 0.5 0.5 0.5 0.5 -9999 -9999 -9999 -9999 -9999 -9999 -9999 -9999 -9999 -9999 -9999 -9999 -9999 -9999 -9999

-9999 -9999 -9999 -9999 -9999 -9999 -9999 -9999 -9999 -9999 -9999 -9999 -9999 -9999 -9999 -9999 -9999 -9999 -9999 -9999 -9999 -9999 -9999 -9999 -9999 -9999 -9999 -9999 -9999 -9999 -9999 -9999 -9999 -9999 -9999 0.1 0.1 0.5 0.5 0.1 0.1 0.1 0.1 0.1 0.1 0.1 0.1 0.1 0.1 0.1 0.1 0.1 0.1 0.1 0.5 -9999 0.5 0.5 0.1 0.1 0.1 0.5 0.5 0.5 0.5 0.5 0.5 -9999 -9999 -9999 -9999 -9999 -9999 -9999 -9999 -9999 -9999 -9999 -9999 -9999 -9999 -9999 -9999

-9999 -9999 -9999 -9999 -9999 -9999 -9999 -9999 -9999 -9999 -9999 -9999 -9999 -9999 -9999 -9999 -9999 -9999 -9999 -9999 -9999 -9999 -9999 -9999 -9999 -9999 -9999 -9999 -9999 -9999 -9999 -9999 -9999 -9999 -9999 0.5 0.1 0.5 0.1 0.5 0.5 0.1 0.1 0.1 0.1 0.1 0.1 0.1 0.1 0.1 0.1 0.1 0.1 0.1 0.5 -9999 0.5 0.5 0.5 0.1 0.1 0.1 0.5 0.5 0.5 0.5 0.1 0.5 -9999 -9999 -9999 -9999 -9999 -9999 -9999 -9999 -9999 -9999 -9999 -9999 -9999 -9999 -9999

-9999 -9999 -9999 -9999 -9999 -9999 -9999 -9999 -9999 -9999 -9999 -9999 -9999 -9999 -9999 -9999 -9999 -9999 -9999 -9999 -9999 -9999 -9999 -9999 -9999 -9999 -9999 -9999 -9999 -9999 -9999 -9999 -9999 -9999 -9999 -9999 0.5 0.5 0.1 0.5 0.1 0.5 0.1 0.1 0.1 0.1 0.1 0.1 0.1 0.1 0.1 0.1 0.5 0.5 0.5 0.5 -9999 0.5 0.5 0.5 0.1 0.5 0.5 0.5 0.5 0.5 0.1 -9999 -9999 -9999 -9999 -9999 -9999 -9999 -9999 -9999 -9999 -9999 -9999 -9999 -9999 -9999 -9999

-9999 -9999 -9999 -9999 -9999 -9999 -9999 -9999 -9999 -9999 -9999 -9999 -9999 -9999 -9999 -9999 -9999 -9999 -9999 -9999 -9999 -9999 -9999 -9999 -9999 -9999 -9999 -9999 -9999 -9999 -9999 -9999 -9999 -9999 -9999 -9999 0.5 0.5 0.5 0.5 0.5 0.1 0.1 0.1 0.1 0.1 0.1 0.1 0.1 0.1 0.1 0.1 0.5 0.5 0.5 0.5 0.5 0.5 0.5 0.5 0.5 0.5 0.5 0.5 0.5 0.5 0.1 -9999 -9999 -9999 -9999 -9999 -9999 -9999 -9999 -9999 -9999 -9999 -9999 -9999 -9999 -9999 -9999

-9999 -9999 -9999 -9999 -9999 -9999 -9999 -9999 -9999 -9999 -9999 -9999 -9999 -9999 -9999 -9999 -9999 -9999 -9999 -9999 -9999 -9999 -9999 -9999 -9999 -9999 -9999 -9999 -9999 -9999 -9999 -9999 -9999 -9999 -9999 -9999 0.1 0.5 0.5 0.1 0.5 0.5 0.1 0.1 0.1 0.1 0.1 0.1 0.1 0.1 0.5 0.5 0.5 0.5 0.5 0.5 0.5 0.5 0.5 0.5 0.5 0.5 0.5 0.5 0.5 0.5 0.1 -9999 -9999 -9999 -9999 -9999 -9999 -9999 -9999 -9999 -9999 -9999 -9999 -9999 -9999 -9999 -9999

-9999 -9999 -9999 -9999 -9999 -9999 -9999 -9999 -9999 -9999 -9999 -9999 -9999 -9999 -9999 -9999 -9999 -9999 -9999 -9999 -9999 -9999 -9999 -9999 -9999 -9999 -9999 -9999 -9999 -9999 -9999 -9999 -9999 -9999 -9999 -9999 0.1 0.5 0.5 0.1 0.5 0.5 0.5 0.1 0.1 0.1 0.1 0.1 0.1 0.1 0.5 0.5 0.5 0.5 0.5 0.1 0.1 0.1 0.1 0.5 0.5 0.5 0.5 0.5 0.5 0.5 0.1 0.5 -9999 -9999 -9999 -9999 -9999 -9999 -9999 -9999 -9999 -9999 -9999 -9999 -9999 -9999 -9999

-9999 -9999 -9999 -9999 -9999 -9999 -9999 -9999 -9999 -9999 -9999 -9999 -9999 -9999 -9999 -9999 -9999 -9999 -9999 -9999 -9999 -9999 -9999 -9999 -9999 -9999 -9999 -9999 -9999 -9999 -9999 -9999 -9999 -9999 -9999 -9999 -9999 0.5 0.5 0.5 0.5 0.1 0.1 0.1 0.1 0.1 0.1 0.1 0.1 0.1 0.1 0.5 0.1 0.5 0.5 0.1 0.1 0.1 0.5 0.1 0.5 -9999 0.5 0.5 0.5 0.1 0.5 0.5 -9999 -9999 -9999 -9999 -9999 -9999 -9999 -9999 -9999 -9999 -9999 -9999 -9999 -9999 -9999

-9999 -9999 -9999 -9999 -9999 -9999 -9999 -9999 -9999 -9999 -9999 -9999 -9999 -9999 -9999 -9999 -9999 -9999 -9999 -9999 -9999 -9999 -9999 -9999 -9999 -9999 -9999 -9999 -9999 -9999 -9999 -9999 -9999 -9999 -9999 -9999 -9999 0.5 0.5 0.1 0.5 0.1 0.1 0.1 0.1 0.1 0.1 0.1 0.5 0.1 0.1 0.1 0.1 0.1 0.1 0.1 0.1 0.5 0.5 0.1 0.5 -9999 0.5 0.5 0.1 0.1 0.1 0.5 0.5 -9999 -9999 -9999 -9999 -9999 -9999 -9999 -9999 -9999 -9999 -9999 -9999 -9999 -9999

-9999 -9999 -9999 -9999 -9999 -9999 -9999 -9999 -9999 -9999 -9999 -9999 -9999 -9999 -9999 -9999 -9999 -9999 -9999 -9999 -9999 -9999 -9999 -9999 -9999 -9999 -9999 -9999 -9999 -9999 -9999 -9999 -9999 -9999 -9999 -9999 -9999 0.5 0.5 0.1 0.5 0.1 0.1 0.1 0.1 0.1 0.1 0.1 0.1 0.1 0.1 0.1 0.1 0.1 0.1 0.1 0.1 0.5 0.5 0.1 0.5 -9999 0.5 0.5 0.1 0.1 0.1 0.1 0.9 -9999 -9999 -9999 -9999 -9999 -9999 -9999 -9999 -9999 -9999 -9999 -9999 -9999 -9999

-9999 -9999 -9999 -9999 -9999 -9999 -9999 -9999 -9999 -9999 -9999 -9999 -9999 -9999 -9999 -9999 -9999 -9999 -9999 -9999 -9999 -9999 -9999 -9999 -9999 -9999 -9999 -9999 -9999 -9999 -9999 -9999 -9999 -9999 -9999 -9999 0.5 0.5 0.5 0.1 0.1 0.1 0.1 0.1 0.1 0.1 0.1 0.1 0.1 0.1 0.1 0.1 0.1 0.1 0.1 0.1 0.5 0.5 0.5 0.1 0.5 -9999 0.5 0.5 0.5 0.1 0.1 0.1 0.9 -9999 -9999 -9999 -9999 -9999 -9999 -9999 -9999 -9999 -9999 -9999 -9999 -9999 -9999

-9999 -9999 -9999 -9999 -9999 -9999 -9999 -9999 -9999 -9999 -9999 -9999 -9999 -9999 -9999 -9999 -9999 -9999 -9999 -9999 -9999 -9999 -9999 -9999 -9999 -9999 -9999 -9999 -9999 -9999 -9999 -9999 -9999 -9999 -9999 -9999 0.5 0.5 0.1 0.1 0.1 0.1 0.1 0.1 0.1 0.1 0.1 0.1 0.1 0.1 0.1 0.1 0.1 0.1 0.1 0.5 0.5 0.5 0.5 0.5 0.5 -9999 0.5 0.1 0.5 0.1 0.1 0.1 0.5 -9999 -9999 -9999 -9999 -9999 -9999 -9999 -9999 -9999 -9999 -9999 -9999 -9999 -9999

-9999 -9999 -9999 -9999 -9999 -9999 -9999 -9999 -9999 -9999 -9999 -9999 -9999 -9999 -9999 -9999 -9999 -9999 -9999 -9999 -9999 -9999 -9999 -9999 -9999 -9999 -9999 -9999 -9999 -9999 -9999 -9999 -9999 -9999 -9999 0.9 0.5 0.1 0.1 0.1 0.1 0.1 0.1 0.1 0.1 0.1 0.1 0.1 0.1 0.1 0.1 0.1 0.1 0.1 0.5 0.5 0.5 0.5 0.5 0.5 0.5 0.5 -9999 0.5 0.5 0.5 0.5 0.1 0.5 -9999 -9999 -9999 -9999 -9999 -9999 -9999 -9999 -9999 -9999 -9999 -9999 -9999 -9999

-9999 -9999 -9999 -9999 -9999 -9999 -9999 -9999 -9999 -9999 -9999 -9999 -9999 -9999 -9999 -9999 -9999 -9999 -9999 -9999 -9999 -9999 -9999 -9999 -9999 -9999 -9999 -9999 -9999 -9999 -9999 -9999 -9999 -9999 -9999 0.5 0.5 0.1 0.1 0.1 0.1 0.1 0.1 0.1 0.1 0.1 0.1 0.1 0.1 0.1 0.1 0.1 0.1 0.1 0.5 0.5 0.5 0.5 0.5 0.5 0.5 0.5 0.5 0.5 0.5 0.5 0.1 0.1 0.9 -9999 -9999 -9999 -9999 -9999 -9999 -9999 -9999 -9999 -9999 -9999 -9999 -9999 -9999

-9999 -9999 -9999 -9999 -9999 -9999 -9999 -9999 -9999 -9999 -9999 -9999 -9999 -9999 -9999 -9999 -9999 -9999 -9999 -9999 -9999 -9999 -9999 -9999 -9999 -9999 -9999 -9999 -9999 -9999 -9999 -9999 -9999 -9999 0.5 0.5 0.5 0.1 0.1 0.1 0.1 0.1 0.1 0.1 0.1 0.1 0.1 0.1 0.1 0.1 0.1 0.1 0.5 0.5 0.5 0.5 0.5 0.5 0.5 0.5 0.5 0.1 0.5 0.5 0.1 0.5 0.5 0.5 -9999 -9999 -9999 -9999 -9999 -9999 -9999 -9999 -9999 -9999 -9999 -9999 -9999 -9999 -9999

-9999 -9999 -9999 -9999 -9999 -9999 -9999 -9999 -9999 -9999 -9999 -9999 -9999 -9999 -9999 -9999 -9999 -9999 -9999 -9999 -9999 -9999 -9999 -9999 -9999 -9999 -9999 -9999 -9999 -9999 -9999 -9999 -9999 -9999 0.5 0.5 0.5 0.1 0.1 0.1 0.1 0.1 0.1 0.1 0.1 0.1 0.1 0.1 0.1 0.1 0.1 0.5 0.5 0.5 0.1 0.5 0.5 0.5 0.5 0.5 0.5 0.1 0.5 0.1 0.1 0.5 0.9 -9999 -9999 -9999 -9999 -9999 -9999 -9999 -9999 -9999 -9999 -9999 -9999 -9999 -9999 -9999 -9999

-9999 -9999 -9999 -9999 -9999 -9999 -9999 -9999 -9999 -9999 -9999 -9999 -9999 -9999 -9999 -9999 -9999 -9999 -9999 -9999 -9999 -9999 -9999 -9999 -9999 -9999 -9999 -9999 -9999 -9999 -9999 -9999 -9999 -9999 -9999 0.5 0.5 0.1 0.1 0.1 0.1 0.1 0.1 0.1 0.1 0.1 0.1 0.1 0.1 0.1 0.1 0.5 0.5 0.1 0.1 0.1 0.1 0.1 0.5 0.5 0.5 0.1 0.1 0.1 0.5 -9999 -9999 -9999 -9999 -9999 -9999 -9999 -9999 -9999 -9999 -9999 -9999 -9999 -9999 -9999 -9999 -9999 -9999

-9999 -9999 -9999 -9999 -9999 -9999 -9999 -9999 -9999 -9999 -9999 -9999 -9999 -9999 -9999 -9999 -9999 -9999 -9999 -9999 -9999 -9999 -9999 -9999 -9999 -9999 -9999 -9999 -9999 -9999 -9999 -9999 -9999 -9999 -9999 0.5 0.5 0.1 0.1 0.1 0.1 0.5 0.1 0.1 0.1 0.1 0.1 0.1 0.1 0.1 0.1 0.1 0.1 0.1 0.1 0.1 0.1 0.5 0.5 0.5 0.5 0.1 0.1 0.1 -9999 -9999 -9999 -9999 -9999 -9999 -9999 -9999 -9999 -9999 -9999 -9999 -9999 -9999 -9999 -9999 -9999 -9999 -9999

-9999 -9999 -9999 -9999 -9999 -9999 -9999 -9999 -9999 -9999 -9999 -9999 -9999 -9999 -9999 -9999 -9999 -9999 -9999 -9999 -9999 -9999 -9999 -9999 -9999 -9999 -9999 -9999 -9999 -9999 -9999 -9999 -9999 -9999 -9999 0.5 0.5 0.5 0.5 0.1 0.1 0.5 0.1 0.1 0.1 0.1 0.1 0.1 0.1 0.1 0.1 0.1 0.1 0.1 0.1 0.5 0.5 0.5 0.5 0.5 0.5 0.1 0.1 -9999 -9999 -9999 -9999 -9999 -9999 -9999 -9999 -9999 -9999 -9999 -9999 -9999 -9999 -9999 -9999 -9999 -9999 -9999 -9999

-9999 -9999 -9999 -9999 -9999 -9999 -9999 -9999 -9999 -9999 -9999 -9999 -9999 -9999 -9999 -9999 -9999 -9999 -9999 -9999 -9999 -9999 -9999 -9999 -9999 -9999 -9999 -9999 -9999 -9999 -9999 -9999 -9999 -9999 -9999 -9999 0.5 0.5 0.5 0.5 0.1 0.1 0.1 0.1 0.1 0.1 0.1 0.1 0.1 0.1 0.1 0.1 0.1 0.5 0.5 0.5 0.5 0.5 0.5 0.5 0.1 0.1 -9999 -9999 -9999 -9999 -9999 -9999 -9999 -9999 -9999 -9999 -9999 -9999 -9999 -9999 -9999 -9999 -9999 -9999 -9999 -9999 -9999

-9999 -9999 -9999 -9999 -9999 -9999 -9999 -9999 -9999 -9999 -9999 -9999 -9999 -9999 -9999 -9999 -9999 -9999 -9999 -9999 -9999 -9999 -9999 -9999 -9999 -9999 -9999 -9999 -9999 -9999 -9999 -9999 -9999 -9999 -9999 -9999 0.5 0.5 0.5 0.5 0.5 0.1 0.1 0.1 0.1 0.1 0.1 0.1 0.1 0.1 0.1 0.1 0.1 0.1 0.1 0.1 0.1 0.1 0.1 0.1 0.1 0.1 -9999 -9999 -9999 -9999 -9999 -9999 -9999 -9999 -9999 -9999 -9999 -9999 -9999 -9999 -9999 -9999 -9999 -9999 -9999 -9999 -9999

-9999 -9999 -9999 -9999 -9999 -9999 -9999 -9999 -9999 -9999 -9999 -9999 -9999 -9999 -9999 -9999 -9999 -9999 -9999 -9999 -9999 -9999 -9999 -9999 -9999 -9999 -9999 -9999 -9999 -9999 -9999 -9999 -9999 -9999 -9999 -9999 -9999 0.5 0.5 0.5 0.5 0.1 0.1 0.1 0.1 0.1 0.1 0.1 0.1 0.1 0.1 0.1 0.1 0.1 0.1 0.5 0.5 0.5 0.1 0.1 0.1 0.1 0.5 -9999 -9999 -9999 -9999 -9999 -9999 -9999 -9999 -9999 -9999 -9999 -9999 -9999 -9999 -9999 -9999 -9999 -9999 -9999 -9999

-9999 -9999 -9999 -9999 -9999 -9999 -9999 -9999 -9999 -9999 -9999 -9999 -9999 -9999 -9999 -9999 -9999 -9999 -9999 -9999 -9999 -9999 -9999 -9999 -9999 -9999 -9999 -9999 -9999 -9999 -9999 -9999 -9999 -9999 -9999 -9999 -9999 -9999 0.5 0.5 0.5 0.1 0.1 0.1 0.1 0.1 0.1 0.1 0.1 0.1 0.1 0.1 0.1 0.1 0.5 0.5 0.5 0.1 0.1 0.1 0.1 0.1 0.5 -9999 -9999 -9999 -9999 -9999 -9999 -9999 -9999 -9999 -9999 -9999 -9999 -9999 -9999 -9999 -9999 -9999 -9999 -9999 -9999

-9999 -9999 -9999 -9999 -9999 -9999 -9999 -9999 -9999 -9999 -9999 -9999 -9999 -9999 -9999 -9999 -9999 -9999 -9999 -9999 -9999 -9999 -9999 -9999 -9999 -9999 -9999 -9999 -9999 -9999 -9999 -9999 -9999 -9999 -9999 -9999 -9999 -9999 0.5 0.5 0.5 0.1 0.1 0.1 0.1 0.1 0.1 0.1 0.1 0.1 0.1 0.1 0.1 0.5 0.5 0.5 0.5 0.5 0.1 0.1 0.1 0.1 0.5 -9999 -9999 -9999 -9999 -9999 -9999 -9999 -9999 -9999 -9999 -9999 -9999 -9999 -9999 -9999 -9999 -9999 -9999 -9999 -9999

-9999 -9999 -9999 -9999 -9999 -9999 -9999 -9999 -9999 -9999 -9999 -9999 -9999 -9999 -9999 -9999 -9999 -9999 -9999 -9999 -9999 -9999 -9999 -9999 -9999 -9999 -9999 -9999 -9999 -9999 -9999 -9999 -9999 -9999 -9999 -9999 -9999 -9999 0.5 0.5 0.5 0.1 0.1 0.1 0.1 0.1 0.1 0.1 0.1 0.1 0.1 0.1 0.1 0.5 0.5 0.5 0.5 0.5 0.5 0.1 0.5 0.1 -9999 -9999 -9999 -9999 -9999 -9999 -9999 -9999 -9999 -9999 -9999 -9999 -9999 -9999 -9999 -9999 -9999 -9999 -9999 -9999 -9999

-9999 -9999 -9999 -9999 -9999 -9999 -9999 -9999 -9999 -9999 -9999 -9999 -9999 -9999 -9999 -9999 -9999 -9999 -9999 -9999 -9999 -9999 -9999 -9999 -9999 -9999 -9999 -9999 -9999 -9999 -9999 -9999 -9999 -9999 -9999 -9999 -9999 -9999 0.5 0.5 0.5 0.1 0.1 0.1 0.1 0.1 0.1 0.1 0.1 0.1 0.1 0.1 0.5 0.5 0.5 0.1 0.5 0.5 0.5 0.5 0.5 -9999 -9999 -9999 -9999 -9999 -9999 -9999 -9999 -9999 -9999 -9999 -9999 -9999 -9999 -9999 -9999 -9999 -9999 -9999 -9999 -9999 -9999

-9999 -9999 -9999 -9999 -9999 -9999 -9999 -9999 -9999 -9999 -9999 -9999 -9999 -9999 -9999 -9999 -9999 -9999 -9999 -9999 -9999 -9999 -9999 -9999 -9999 -9999 -9999 -9999 -9999 -9999 -9999 -9999 -9999 -9999 -9999 -9999 -9999 -9999 0.5 0.5 0.5 0.5 0.5 0.1 0.1 0.1 0.1 0.1 0.1 0.1 0.1 0.1 0.1 0.1 0.1 0.1 0.5 0.5 0.5 -9999 -9999 -9999 -9999 -9999 -9999 -9999 -9999 -9999 -9999 -9999 -9999 -9999 -9999 -9999 -9999 -9999 -9999 -9999 -9999 -9999 -9999 -9999 -9999

-9999 -9999 -9999 -9999 -9999 -9999 -9999 -9999 -9999 -9999 -9999 -9999 -9999 -9999 -9999 -9999 -9999 -9999 -9999 -9999 -9999 -9999 -9999 -9999 -9999 -9999 -9999 -9999 -9999 -9999 -9999 -9999 -9999 -9999 -9999 -9999 -9999 -9999 -9999 0.5 0.5 0.5 0.5 0.1 0.1 0.1 0.1 0.5 0.1 0.1 0.1 0.1 0.1 0.1 0.1 0.5 0.5 0.5 0.5 0.5 -9999 -9999 -9999 -9999 -9999 -9999 -9999 -9999 -9999 -9999 -9999 -9999 -9999 -9999 -9999 -9999 -9999 -9999 -9999 -9999 -9999 -9999 -9999

-9999 -9999 -9999 -9999 -9999 -9999 -9999 -9999 -9999 -9999 -9999 -9999 -9999 -9999 -9999 -9999 -9999 -9999 -9999 -9999 -9999 -9999 -9999 -9999 -9999 -9999 -9999 -9999 -9999 -9999 -9999 -9999 -9999 -9999 -9999 -9999 -9999 -9999 -9999 0.5 0.5 0.5 0.5 0.5 0.1 0.1 0.1 0.5 0.5 0.1 0.1 0.1 0.1 0.1 0.5 0.5 0.5 0.5 0.5 -9999 -9999 -9999 -9999 -9999 -9999 -9999 -9999 -9999 -9999 -9999 -9999 -9999 -9999 -9999 -9999 -9999 -9999 -9999 -9999 -9999 -9999 -9999 -9999

-9999 -9999 -9999 -9999 -9999 -9999 -9999 -9999 -9999 -9999 -9999 -9999 -9999 -9999 -9999 -9999 -9999 -9999 -9999 -9999 -9999 -9999 -9999 -9999 -9999 -9999 -9999 -9999 -9999 -9999 -9999 -9999 -9999 -9999 -9999 -9999 -9999 -9999 -9999 -9999 0.5 0.5 0.5 0.5 0.1 0.1 0.1 0.5 0.1 0.1 0.1 0.1 0.1 0.5 0.5 0.5 0.5 0.5 0.9 -9999 -9999 -9999 -9999 -9999 -9999 -9999 -9999 -9999 -9999 -9999 -9999 -9999 -9999 -9999 -9999 -9999 -9999 -9999 -9999 -9999 -9999 -9999 -9999

-9999 -9999 -9999 -9999 -9999 -9999 -9999 -9999 -9999 -9999 -9999 -9999 -9999 -9999 -9999 -9999 -9999 -9999 -9999 -9999 -9999 -9999 -9999 -9999 -9999 -9999 -9999 -9999 -9999 -9999 -9999 -9999 -9999 -9999 -9999 -9999 -9999 -9999 -9999 -9999 0.5 0.5 0.5 0.1 0.1 0.1 0.1 0.1 0.1 0.1 0.1 0.1 0.5 0.5 0.5 0.5 0.5 0.9 -9999 -9999 -9999 -9999 -9999 -9999 -9999 -9999 -9999 -9999 -9999 -9999 -9999 -9999 -9999 -9999 -9999 -9999 -9999 -9999 -9999 -9999 -9999 -9999 -9999

-9999 -9999 -9999 -9999 -9999 -9999 -9999 -9999 -9999 -9999 -9999 -9999 -9999 -9999 -9999 -9999 -9999 -9999 -9999 -9999 -9999 -9999 -9999 -9999 -9999 -9999 -9999 -9999 -9999 -9999 -9999 -9999 -9999 -9999 -9999 -9999 -9999 -9999 -9999 -9999 -9999 0.5 0.5 0.5 0.1 0.1 0.5 0.1 0.1 0.1 0.5 0.5 0.5 0.5 0.5 0.5 0.9 -9999 -9999 -9999 -9999 -9999 -9999 -9999 -9999 -9999 -9999 -9999 -9999 -9999 -9999 -9999 -9999 -9999 -9999 -9999 -9999 -9999 -9999 -9999 -9999 -9999 -9999

-9999 -9999 -9999 -9999 -9999 -9999 -9999 -9999 -9999 -9999 -9999 -9999 -9999 -9999 -9999 -9999 -9999 -9999 -9999 -9999 -9999 -9999 -9999 -9999 -9999 -9999 -9999 -9999 -9999 -9999 -9999 -9999 -9999 -9999 -9999 -9999 -9999 -9999 -9999 -9999 -9999 -9999 0.5 0.5 0.5 0.5 0.5 0.5 0.5 0.5 0.5 0.5 0.5 0.5 0.5 0.9 0.9 -9999 -9999 -9999 -9999 -9999 -9999 -9999 -9999 -9999 -9999 -9999 -9999 -9999 -9999 -9999 -9999 -9999 -9999 -9999 -9999 -9999 -9999 -9999 -9999 -9999 -9999

-9999 -9999 -9999 -9999 -9999 -9999 -9999 -9999 -9999 -9999 -9999 -9999 -9999 -9999 -9999 -9999 -9999 -9999 -9999 -9999 -9999 -9999 -9999 -9999 -9999 -9999 -9999 -9999 -9999 -9999 -9999 -9999 -9999 -9999 -9999 -9999 -9999 -9999 -9999 -9999 -9999 -9999 0.5 0.5 0.5 0.5 0.5 0.5 0.5 0.5 0.5 0.5 0.5 0.5 0.9 0.9 -9999 -9999 -9999 -9999 -9999 -9999 -9999 -9999 -9999 -9999 -9999 -9999 -9999 -9999 -9999 -9999 -9999 -9999 -9999 -9999 -9999 -9999 -9999 -9999 -9999 -9999 -9999

-9999 -9999 -9999 -9999 -9999 -9999 -9999 -9999 -9999 -9999 -9999 -9999 -9999 -9999 -9999 -9999 -9999 -9999 -9999 -9999 -9999 -9999 -9999 -9999 -9999 -9999 -9999 -9999 -9999 -9999 -9999 -9999 -9999 -9999 -9999 -9999 -9999 -9999 -9999 -9999 -9999 -9999 0.5 0.5 0.5 0.5 0.5 0.5 0.5 0.5 0.5 0.5 0.5 0.9 0.9 -9999 -9999 -9999 -9999 -9999 -9999 -9999 -9999 -9999 -9999 -9999 -9999 -9999 -9999 -9999 -9999 -9999 -9999 -9999 -9999 -9999 -9999 -9999 -9999 -9999 -9999 -9999 -9999

-9999 -9999 -9999 -9999 -9999 -9999 -9999 -9999 -9999 -9999 -9999 -9999 -9999 -9999 -9999 -9999 -9999 -9999 -9999 -9999 -9999 -9999 -9999 -9999 -9999 -9999 -9999 -9999 -9999 -9999 -9999 -9999 -9999 -9999 -9999 -9999 -9999 -9999 -9999 -9999 -9999 -9999 0.5 0.5 0.5 0.5 0.5 0.5 0.5 0.5 0.5 0.9 -9999 -9999 -9999 -9999 -9999 -9999 -9999 -9999 -9999 -9999 -9999 -9999 -9999 -9999 -9999 -9999 -9999 -9999 -9999 -9999 -9999 -9999 -9999 -9999 -9999 -9999 -9999 -9999 -9999 -9999 -9999

-9999 -9999 -9999 -9999 -9999 -9999 -9999 -9999 -9999 -9999 -9999 -9999 -9999 -9999 -9999 -9999 -9999 -9999 -9999 -9999 -9999 -9999 -9999 -9999 -9999 -9999 -9999 -9999 -9999 -9999 -9999 -9999 -9999 -9999 -9999 -9999 -9999 -9999 -9999 -9999 -9999 -9999 0.5 0.5 0.1 0.1 0.1 -9999 -9999 -9999 -9999 -9999 -9999 -9999 -9999 -9999 -9999 -9999 -9999 -9999 -9999 -9999 -9999 -9999 -9999 -9999 -9999 -9999 -9999 -9999 -9999 -9999 -9999 -9999 -9999 -9999 -9999 -9999 -9999 -9999 -9999 -9999 -9999
